# How do we provide good mealtime care for people with dementia living in care homes? A systematic review of carer–resident interactions

**DOI:** 10.1177/14713012211002041

**Published:** 2021-04-07

**Authors:** James Faraday, Clare Abley, Fiona Beyer, Catherine Exley, Paula Moynihan, Joanne M Patterson

**Affiliations:** Population Health Sciences Institute, 5994 Newcastle University, UK; 5983 The Newcastle upon Tyne Hospitals NHS Foundation Trust, UK; 5983 The Newcastle upon Tyne Hospitals NHS Foundation Trust, UK; Population Health Sciences Institute, 5994 Newcastle University, UK; Population Health Sciences Institute, 5994 Newcastle University, UK; Faculty of Medical Sciences, 5994 Newcastle University, UK; Faculty of Health and Medical Sciences, 50072 The University of Adelaide, Australia; Faculty of Medical Sciences, 5994 Newcastle University, UK; School of Health Sciences, 4591 The University of Liverpool, UK

**Keywords:** dementia, meals, long-term care, caregivers, systematic review

## Abstract

People with dementia who live in care homes often depend on care home staff for help with eating and drinking. It is essential that care home staff have the skills and support they need to provide good care at mealtimes. Good mealtime care may improve quality of life for residents, and reduce hospital admissions. The aim of this systematic review was to identify good practice in mealtime care for people with dementia living in care homes, by focusing on carer-resident interactions at mealtimes. Robust systematic review methods were followed. Seven databases were searched: AgeLine, BNI, CENTRAL, CINAHL, MEDLINE, PsycINFO and Web of Science. Titles, abstracts, and full texts were screened independently by two reviewers, and study quality was assessed with Joanna Briggs Institute tools. Narrative synthesis was used to analyse quantitative and qualitative evidence in parallel. Data were interrogated to identify thematic categories of carer-resident interaction. The synthesis process was undertaken by one reviewer, and discussed throughout with other reviewers for cross-checking. After title/abstract and full-text screening, 18 studies were included. Some studies assessed mealtime care interventions, others investigated factors contributing to oral intake, whilst others explored the mealtime experience. The synthesis identified four categories of carer-resident interaction important to mealtime care: Social connection, Tailored care, Empowering the resident, and Responding to food refusal. Each of the categories has echoes in related literature, and provides promising directions for future research. They merit further consideration, as new interventions are developed to improve mealtime care for this population.

## Introduction

Dementia describes a set of symptoms including concentration and memory problems, changes in mood and behaviour and problems with communication and reasoning (Alzheimer’s Society, 2014). It is an increasingly prevalent condition; by 2025, there are expected to be one million people with dementia in the United Kingdom (Department of Health (UK), 2015).

Swallowing difficulties (dysphagia) may occur in people with dementia due to impaired physiology (such as a delayed swallow reflex) and/or due to behavioural issues (such as eating and drinking too quickly) (Alagiakrishnan et al., 2013). This can result in pulmonary aspiration (entry of food/drink into the larynx and lower respiratory tract), which in turn can lead directly to asphyxiation or pneumonia (Torres et al., 2013).

Mealtime difficulties in people with dementia are not limited to dysphagia; other problems at mealtimes are prevalent (Kai et al., 2015). These include difficulty recognising food and drink (Amella, 2002), problems using cutlery (Social Care Institute for Excellence, 2015) and changes in appetite and preferences (Ikeda et al., 2002). Such difficulties have potentially serious consequences. Inadequate oral intake may result in undernutrition and dehydration and therefore reduced quality of life, more frequent hospital admissions and increased risk of morbidity and mortality (Abbott et al., 2013).

In addition, eating and drinking are fundamental human activities, with significant social and emotional associations (Brush & Calkins, 2008; Burges Watson et al., 2018). The psychosocial aspect of mealtimes is important and may impact on food intake and quality of life (Fetherstonhaugh et al., 2019; Keller, 2016). Difficulties at mealtimes can lead to anxiety, depression and isolation (Ney et al., 2009). For all these reasons, mealtime difficulties can be distressing for people with dementia and for those who provide their care (Pasman et al., 2003).

Mealtime difficulties are particularly prevalent among people with dementia in long-term care settings (Prince et al., 2014). In the United Kingdom, United States and Australia, it has been estimated that more than half of all care home residents have dementia (Alzheimer’s Society, 2014; Australian Institute of Health and Welfare, 2012; Harris-Kojetin et al., 2016). In care homes, it is staff who provide personal care to people with dementia – including at mealtimes. Typically, this is staff in a carer role (whether named as carer, care assistant, certified nursing assistant, auxiliary, care aide, and so on, depending on country). Qualified nursing staff may also provide this personal care at mealtimes. Through their interaction with residents, care home staff are responsible for facilitating oral intake which is safe, adequate and enjoyable (Health Education England, 2015). They may do this by providing physical assistance with eating/drinking (Abdelhamid et al., 2016; Mann et al., 2019). They may also do this through prompting and supervision at mealtimes and responding to signs of dysphagia to minimise risk of aspiration. For the purposes of this study, we will refer to all activity of this kind as ‘mealtime care’.

Good mealtime care for people with dementia living in care homes can improve their quality of life (Evans et al., 2009), provide greater reassurance for their families (Alzheimer’s Society, 2013; Hanson et al., 2013) and may reduce hospital admissions (Richardson, 2015). However, evidence has shown that the quality of mealtime care for this population is variable (Aselage et al., 2011). The aim of this systematic review is to identify good practice in mealtime care for people with dementia living in care homes, by focusing on carer–resident interactions at mealtimes.

## Methods

### Registration of the review

The protocol for this review was registered on PROSPERO (CRD42018114533).

### Eligibility criteria

The following eligibility criteria were used for study selection:

1. The population was people with dementia living in care homes. Dementia could be of any type and stage. If a study included participants with various clinical diagnoses, it was necessary for the majority of participants to have dementia and for data on those participants to be presented separately from those with other diagnoses.
2. The phenomenon of interest was mealtime care. Mealtime care was defined as ‘interactions occurring between care staff and residents at mealtimes, which may promote safe, adequate and/or enjoyable oral intake’. Activities outside of the direct control of care staff were excluded (e.g. recommendation of percutaneous endoscopic gastrostomy tube, oral nutritional supplements and specialist training programmes targeted at people with dementia, such as Montessori, and spaced retrieval therapy). Studies focusing on assessment of mealtime difficulties were also excluded.
3. The publication types were peer-reviewed primary studies of any research design (quantitative, qualitative or mixed methods). For practical reasons, sources unavailable in English were excluded from the review. There was no limitation on the date of studies, to capture as many studies as possible which met the eligibility criteria.

### Search strategy

Seven databases were chosen to provide comprehensive and relevant multidisciplinary coverage: AgeLine, BNI, CENTRAL, CINAHL, MEDLINE, PsycINFO and Web of Science. Search strings suitable for each database were devised; these comprised the categories ‘dementia’, ‘mealtimes’ and ‘care’ and used both free-text and index terms. Thesaurus headings were translated as appropriate between databases. An example search strategy, for MEDLINE, is presented in Supplemental Appendix A. Databases were searched from inception to May 2020.

Results were downloaded into EndNote^©^. Titles and abstracts of retrieved studies were screened independently by two reviewers, using the online tool Rayyan (http://rayyan.qcri.org). Any discrepancies were resolved via discussion. Full texts of remaining studies were then screened independently by two reviewers. Again, discrepancies were resolved via discussion, with recourse to a third reviewer as needed. Reasons for exclusion were noted.

In addition to the database search, studies were sought by other means. Relevant non-indexed journals were hand-searched, from inception to present. These were the Journal of Nursing Home Research, the Journal of Aging and Long-Term Care and the Journal of Long-Term Care. Reference lists of related systematic reviews were searched. Experts in the field were contacted for recommendations.

### Data extraction

Separate data extraction forms were designed for quantitative and qualitative studies, with fields chosen to capture all necessary information. Data were extracted on study characteristics, outcomes and results. For qualitative studies, verbatim reports of findings were extracted. One reviewer carried out data extraction, and a second reviewer checked the data against the original papers to ensure there were no erroneous or missing data.

### Critical appraisal

Peer-reviewed critical appraisal tools published by the Joanna Briggs Institute (JBI) were used to critically appraise the studies (Aromataris & Munn, 2017). The tools used were as follows: Checklist for Analytical Cross Sectional Studies, Checklist for Quasi-Experimental Studies, Checklist for Randomised Controlled Trials and Checklist for Qualitative Research. Two reviewers critically appraised all studies independently. Discrepancies were resolved via discussion, with recourse to a third reviewer as needed.

### Data synthesis

A convergent synthesis design was used: quantitative and qualitative evidence was collected and analysed in parallel (Hong et al., 2017). Integration occurred at the level of the extracted data so that studies were analysed using the same synthesis method. The chosen method of analysis was Narrative Synthesis (Popay et al., 2006). In order to construct a common rubric for synthesis of quantitative and qualitative data, a textual summary of results was produced for each study. Verbatim extracts from the study reports were used, including principle findings. Data were interrogated to identify thematic categories of carer–resident interaction. Principles of constant comparative method were used (Glaser, 1965), which involved reading and re-reading data to search for emerging categories (Burnard et al., 2008). The synthesis process was undertaken by one reviewer, with regular discussion with other reviewers in order to cross-check the analysis.

## Results

**Figure 1.**
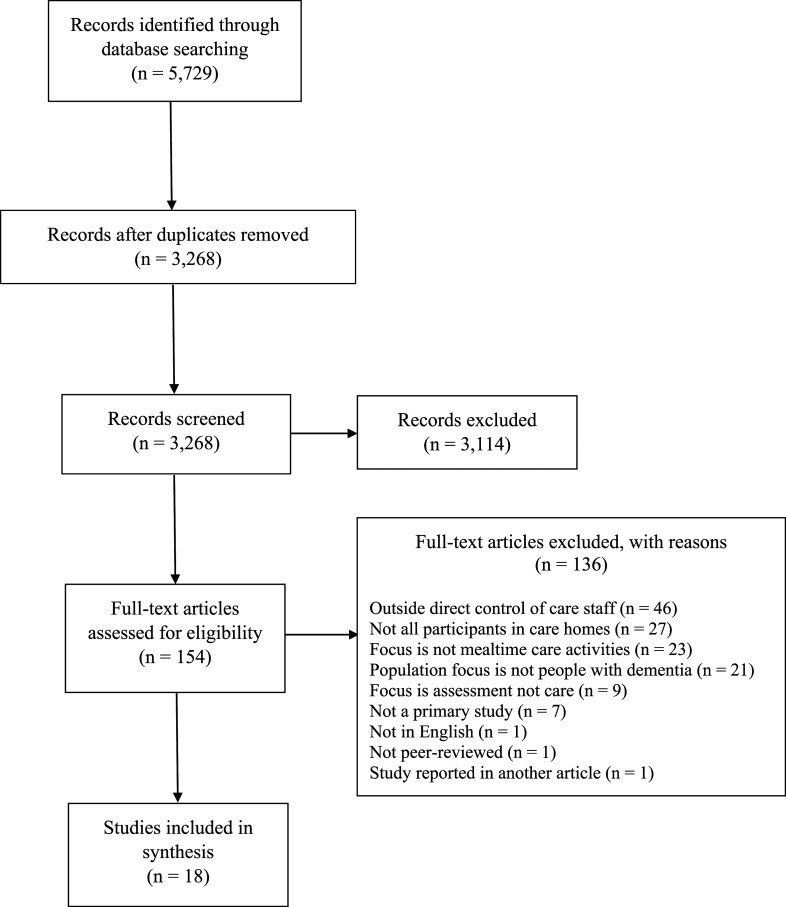
PRISMA flow diagram of study selection process.

The initial database search retrieved 5729 articles. Reference management software was used to remove duplicates, leaving 3268 articles. Title/abstract screening resulted in 680 articles. Of these, 526 were excluded because they were ineligible due to language, availability, publication type or because of duplication undetected by the software. The remaining 154 articles were full-text screened, with 136 excluded at this stage (see Supplemental Appendix B for a full list of these articles and reasons for exclusion). This left 18 articles which reported eligible studies. Hand searches and other lines of enquiry did not yield any additional eligible studies (see Figure 1 for a PRISMA flow diagram summarising the study selection process) (Moher et al., 2009).

### Study characteristics

Study characteristics are presented in Table 1 and summarised here. Nine different countries are represented in the studies: Australia, Canada, Italy, New Zealand, Sweden, Taiwan, the Netherlands, United Kingdom and United States. All of the studies took place in care homes, with the exception of Murphy et al. (2017), where a neutral venue was used for focus groups and interviews. 10 studies recruited residents and care home staff as participants; four studies recruited only residents; four studies recruited only care home staff.

**Table 1.** Study characteristics.

Study	Setting and sample	Design and methods	Intervention/exposure/phenomenon of interest
*Quantitative studies*			
Suski and Nielsen (1989)	*Setting:* Long-term care facility (n = 1), United States.	*Design:* Quantitative: Cross-sectional.	*Exposure:* Time of day; type of food; and type of assistance.
	*Sample:* Residents with Alzheimer’s disease (n = 19).	*Methods:* Food intake recorded for a study group on 3 consecutive days at all meals. Factors that could affect optimal intake, that is the time of day and types of food and assistance, were recorded and evaluated.	
Coyne and Hoskins (1997)	*Setting:* Dementia unit in skilled nursing facility (n = 1), United States.	*Design:* Quantitative: Randomised controlled trial.	*Intervention:* Directed verbal prompt and positive reinforcement. Prompts for solid food: ‘Pick up the [name of utensil]’; ‘Put the food on the [name of utensil]’; ‘Move the food to your mouth’; ‘Put the food in your mouth’; ‘Chew the food’; ‘Swallow the food’. Positive reinforcement: ‘Correct’; ‘That’s right’; ‘Good’.
	*Sample:* Residents with dementia (n = 24).	*Methods:* Experimental group received intervention; control group received usual care. Level of eating independence (LEI) measured at 3 daily meals across 3 time points (1 pretest and 2 post-test).	
Amella (1999)	*Setting:* Nursing home (n = 1), United States.	*Design:* Quantitative: Cross-sectional.	*Exposure:* Interaction between resident and carer; carer empathy; and carer perceived power.
	*Sample:* Residents with dementia (n = 53) and nursing staff (n = 53).	*Methods:* Observations of resident/staff dyads at mealtimes. Interaction, food/fluid intake, and time to complete meal measured. Carer empathy/power measured via questionnaire post-meals.	
Engström and Hammar (2012)	*Setting:* Municipal nursing home (n = 1), Sweden.	*Design:* Quantitative: Quasi-experimental.	*Intervention:* Carers instructed to hum sing-along songs, children’s songs and popular songs from the early part of the 20th century.
	*Sample:* Residents with severe dementia (n = 2).	*Methods:* Each resident video observed at mealtimes once per week over 5 consecutive weeks. Weeks 1 and 2: usual care. Weeks 3 and 4: intervention. Week 5: usual care. Eating behaviour and food intake measured at baseline (x2), intervention (x2) and return to baseline condition (x1).	
Batchelor-Murphy et al. (2017)	*Setting:* Nursing homes (n = 11), United States.	*Design:* Quantitative: Randomised cross-over study (within subjects).	*Intervention:* Three techniques for eating assistance: direct hand; over hand; and under hand. These were defined as follows: direct hand – carer holds utensil, no active involvement from resident; over hand – care puts hand over resident’s hand to assist them; under-hand – care holds utensil and places hand under resident’s hand.
	*Sample:* Residents with dementia (n=30).	*Methods:* Residents received the three interventions in different sequences over 6 consecutive meals. Time providing eating assistance, meal intake, eating behaviours measured.	
*Qualitative studies*			
Van Ort and Phillips (1992)	*Setting:* Special dementia care unit of long-term care facility (n = 1), United States.	*Design:* Qualitative.	*Phenomenon of interest:* Nursing activities, resident behaviours and interactions during mealtimes.
	*Sample:* Residents (n = 10); nursing assistants, licensed practical nurses and registered nurses (n = 11).	*Methods:* Data collection focused on mealtime interactions between carers and residents in the dayroom. Data were videotaped, allowing detailed systematic observation of events. Open coding was used to devise categories of behaviour.	
Kayser-Jones and Schell (1997a)	*Setting:* Nursing homes (n = 2), United States.	*Design:* Qualitative.	*Phenomenon of interest:* Mealtime experience of cognitively impaired resident in nursing home.
	*Sample:* Resident with dementia (n = 1) and nursing staff (n = not stated).	*Methods:* Participant observations and interviews. Event analysis.	
Kayser-Jones & Schell (1997b)	*Setting:* Special care unit for dementia patients (n = 1), United States.	*Design:* Qualitative.	*Phenomenon of interest:* Effect of staffing on the mealtime experience of residents.
	*Sample:* Residents (n = 100); nursing staff (n = 50); physicians (n = 36) and family members (n = 50).	*Methods:* Participant observations and in-depth interviews. Event analysis.	
Pierson (1999)	*Setting:* Long-term care facility (n = 1), United States.	*Design:* Qualitative.	*Phenomenon of interest:* The self-organizing activities and unspoken knowledge of nursing assistants employed in assisting residents with dementia to eat.
	*Sample:* Nursing assistants (n = not stated).	*Methods:* Participant observation of 12 mealtimes across 3 dining areas. Review of residents’ daily food records. Analysis proceeded concurrently with data collection.	
De Bellis et al. (2003)	*Setting:* Residential care facility (n = 1), Australia. *Sample:* Residents with dementia (n = 24) and nursing and personal care staff (n = not stated).	*Design:* Qualitative*.	*Phenomenon of interest:* Issues associated with oral intake of food and fluids from perspective of the resident and/or carer.
		*Methods:* Non-participant observation of each resident and the person assisting with eating and drinking, for one mealtime. Qualitative analysis and extrapolation of themes or issues.	
Pasman et al. (2003)	*Setting:* Nursing homes (n = 2), the Netherlands.	*Design:* Qualitative.	*Phenomenon of interest:* The nature of the problems faced by nurses when assisting nursing home patients with severe dementia at mealtimes and how they deal with these problems.
	*Sample:* Residents (n = 60) and nurses (n = 46).	*Methods:* Participant observation, formal interviews and access to medical records. Constant comparison analysis.	
Gibbs-Ward and Keller (2005)	*Setting:* Special care units, long-term care facilities (n = 2), Canada.	*Design:* Qualitative.	*Phenomenon of interest:* Mealtime care for people with dementia in long-term care facilities.
	*Sample:* Residents (n = 20); health care aides (n = 18); registered nurse (n = 1); registered practical nurses (n = 4) and registered dieticians (n = 8).	*Methods:* Observations of 9 meals during 3 to 4 non-consecutive days, using meal observation form. Semi-structured interviews with key informants; questions informed by observations. Constant comparison analysis.	
Chang and Roberts (2008)	*Setting:* Nursing home (n = 1), Taiwan.	*Design:* Qualitative**.	*Phenomena of interest:* Eating difficulties; eating assistance strategies; interactions during eating assistance; mealtime environment.
	*Sample:* Residents with dementia (n = 48) and nursing assistants (n = 31).	*Methods:* Observations of mealtimes. Interviews with nursing assistants on strategies used to address eating difficulties. Content analysis.	
Hung and Chaudhury (2011)	*Setting:* Long-term care facilities (n = 2), Canada.	*Design:* Qualitative.	*Phenomenon of interest:* Mealtime care for people with dementia in long-term care facilities.
	*Sample:* Residents with dementia (n = 20); care aides (n = 4); nurses (n = 2) and dieticians (n = 2).	*Methods:* Participant observations, conversational interviews with residents, focus groups with care home staff, review of documents. Integrated deductive–inductive approach to data analysis.	
Nell et al. (2016)	*Setting:* Specialised Dementia Care Units (n = 2), New Zealand.	*Design:* Qualitative.	*Phenomenon of interest:* Perceptions of staff caregivers regarding factors affecting optimal nutrition/hydration.
	*Sample:* Staff caregivers (n = 11)	*Methods:* Semi-structured interviews. General inductive five-step approach to analysis.	
Murphy et al. (2017)	*Setting:* Neutral venue (n = 1), United Kingdom	*Design:* Qualitative.	*Phenomenon of interest:* Complex nutritional problems associated with eating and drinking for people with dementia.
	*Sample:* Care staff (n = 30); family carers (n = 8); dietitians (n = 3) and speech and language therapists (n = 9).	*Methods:* Semi-structured interviews and focus groups. Thematic analysis.	
Palese et al. (2018)	*Setting:* Nursing homes (n = 13), Italy.	*Design:* Qualitative.	*Phenomenon of interest:* Perceived effectiveness of interventions to maintain eating independence among older people with dementia who live in nursing homes.
	*Sample:* Care staff (n = 54).	*Methods:* Observations of mealtimes and focus groups. Qualitative content analysis.	
Driessen and Ibáñez Martín (2020)	*Setting:* Residential care homes (n = 3), the Netherlands.	*Design:* Qualitative.	*Phenomenon of interest:* How carers attend to differences in providing mealtime care.
	*Sample:* Residents (n = 100 approx.) and care professionals (n = 60 approx.).	*Methods:* Participant observation, informal conversations and interviews. Method of analysis not stated.	

*De Bellis et al. (2003) reported qualitative data from a mixed methods study.

**Chang and Roberts (2008) reported a mixed methods study. The quantitative data focused on eating difficulties, while the qualitative data investigated mealtime care. For this reason, only the qualitative data from this study was included in this review.

The studies were varied in design. Two studies were randomised controlled trials (Batchelor-Murphy et al., 2017; Coyne & Hoskins, 1997); one was a quasi-experimental study (Engström & Hammar, 2012); two were cross-sectional studies (Amella, 1999; Suski & Nielsen, 1989). Eleven studies were qualitative. Of these, nine included observation of mealtimes in their data collection (Driessen & Ibáñez Martín, 2020; Gibbs-Ward & Keller, 2005; Hung & Chaudhury, 2011; Kayser-Jones & Schell, 1997a, 1997b; Palese et al., 2018; Pasman et al., 2003; Pierson, 1999; Van Ort & Phillips, 1992) and two used focus groups and/or interviews only (Murphy et al., 2017; Nell et al., 2016). Two studies used mixed methods: Chang and Roberts (2008) and De Bellis et al. (2003). Both of these conducted cross-sectional studies and collected qualitative data. De Bellis et al. (2003) reported only the qualitative data. In Chang and Roberts (2008), the quantitative data focused on residents’ eating difficulty, while the qualitative data investigated mealtime care – for this reason, only the qualitative data from this study were included here. Thus, these two studies are bracketed with the qualitative studies in the review.

The three experimental/quasi-experimental studies assessed the effectiveness of a care staff intervention at mealtimes. Batchelor-Murphy et al. (2017) compared three techniques for eating assistance (direct hand, over hand and under hand), by using the Edinburgh Feeding Evaluation in Dementia scale to assess eating and eating assistance behaviours. Coyne and Hoskins (1997) assessed the efficacy of directed verbal prompts and positive reinforcement, using their own Level of Eating Independence scale. Engström and Hammar (2012) assessed the effect of carers humming during mealtimes, measuring the amount of food/liquid consumed in grams.

The two cross-sectional studies investigated the association between quantity of oral intake and possible influencing factors. Amella (1999) used weight to measure proportion of food consumed and assessed the influence on this of interaction between resident and carer, carer empathy and carers’ level of perceived power (these were measured using, respectively, the Interaction Behaviour Measure, the Interpersonal Reactivity Index and the Fundamental Interpersonal Relations Orientation – Behavior). Suski and Nielsen (1989) used researcher rating of proportion of meal consumed as the outcome measure, in considering the impact of time of day, type of food and technique for eating assistance.

The 13 studies reporting qualitative data explored mealtimes from various perspectives. De Bellis et al. (2003), Kayser-Jones and Schell (1997b), Nell et al. (2016), Palese et al. (2018), Pasman et al. (2003), and Pierson (1999) investigated care staff’s perceptions of, and approaches to, mealtime care. Gibbs-Ward and Keller (2005) and Murphy et al. (2017) used qualitative data to develop a conceptual understanding of mealtime care. Kayser-Jones and Schell (1997a) and Van Ort and Phillips (1992) described and analysed carer strategies to assist residents at mealtimes. Hung and Chaudhury (2011) explored the concept of personhood (see Kitwood, 1997) in the context of mealtimes, with particular reference to the influence of care staff activity. Chang and Roberts (2008) investigated mealtime difficulties, carer strategies and mealtime environment. Driessen and Ibáñez Martín (2020) considered how mealtime care is tailored to address differences in residents.

### Study quality

Detailed results of the critical appraisal are presented in Supplementary Tables A (quantitative studies) and B (qualitative studies) with the online version of this article. The methodological quality of included studies was varied. The highest-scoring quantitative study (Batchelor-Murphy et al., 2017) was rated Yes for all applicable criteria, except for one criterion which was rated Unclear. The lowest-scoring quantitative study (Engström & Hammar, 2012) was rated No for three applicable criteria. The other quantitative studies were all rated No for one applicable criterion. The failed criteria were different in each case and included the following: ‘Were the outcomes measured in a valid and reliable way?’; ‘were confounding factors identified?’ and ‘were treatment groups similar at baseline?’ The highest-scoring qualitative study (Gibbs-Ward & Keller, 2005) met all criteria. The lowest-scoring qualitative study (Chang & Roberts, 2008) was rated No for three criteria and Unclear for six others. The other qualitative studies achieved a range of scores in between. The most common failed criterion amongst the qualitative studies was ‘is the influence of the researcher on the research, and vice versa, addressed?’ The impact of study quality on the review synthesis is discussed in more detail below.

### Synthesis of study findings

The findings of individual studies are presented in Table 2. Using narrative synthesis, these findings were analysed to identify thematic categories of carer–resident interaction. Four broad categories of carer–resident interaction were identified: *Social connection*, *Tailored care, Empowering the resident* and *Responding to food refusal*. These are presented alongside relevant study findings in Table 3 and described below.

**Table 2.** Study findings.

Study	Findings
*Quantitative studies*	
Suski and Nielsen (1989)	Four major factors emerged that promoted optimal intake: using skilful techniques to assist eating, selecting appropriate food consistency, providing adequate time in which to assist eating and capitalizing on the midday meal when cognitive abilities were at their peak.
Coyne and Hoskins (1997)	Significant difference between experimental and control groups for both solid and liquid food on task performance. For solid food: experimental group grand mean = 16.6; control group grand mean = 13.1, F (1,22) = 7.78, p= 0.011. Significant interaction when comparing pretest to first and second post-tests, t = 2.38, p = 0.026. For liquid food: experimental group grand mean = 13.8; control group grand mean = 11.4, F (1,22) = 8.90, p= 0.007. Significant interaction when comparing pretest to first and second post-tests, t = 2.52, p = 0.019.
Amella (1999)	Quality of reciprocal relationship between resident and carer significantly and positively related to proportion of food consumed (R^2^ = .40; F_6,46_ = 5.13; P = .0004). Willingness of carer to let another control their behaviour positively correlated to proportion of food consumed (r = .29; P = .024).
Engström and Hammar (2012)	First resident: negative eating behaviours (as measured by the Edinburgh Feeding Evaluation in Dementia (EdFED) scale) decreased during the humming and increased during the follow-up (Baseline#1 = 14, Baseline#2 = 14, Intervention#1 = 6, Intervention#2 = 11, Follow-up = 16); total oral intake (liquid and meal) increased during intervention. Second resident: negative eating behaviour scores from baseline observations were higher than those recorded during the intervention sessions (Baseline#1 = 8, Baseline#2 = 16, Intervention#1 = 9, Intervention#2 = 6, Follow-up = 5); total oral intake (liquid and meal) decreased during intervention.
Batchelor-Murphy et al. (2017)	Under hand eating assistance technique reduced eating behaviours and promoted meal intake at same level as direct hand, while requiring no additional time to implement. Eating assistance technique had a significant effect on eating behaviours as measured by EdFED total scores per meal (P = .025). The mean total score per meal for OH (8.3, SD 1.8) was significantly higher relative to DH (8.0, SD 1.8, P = .041, Cohen d = 0.17, small effect) and UH (7.7, SD 1.8), P = .001, Cohen d = 0.33, medium effect). Eating assistance technique had a significant effect on percent meal intake per meal based on tray weight (P = .023), with the mean percent meal intake significantly higher for DH (67%, SD 15.2) and UH (65%, SD 15.0) when compared to OH (59.9%, SD 15.1) P < .001 and .001, respectively). Findings suggest that use of each eating assistance technique should be considered within context of the residents’ functional ability, energy level and individual preferences, any of which may vary on a day-to-day, meal-to-meal basis.
*Qualitative studies*	
Van Ort and Phillips (1992)	Three categories were identified which were associated with the resident’s acceptance or rejection of food. These were behaviours that elicit functional eating (visual, tactile and verbal cues were the means used by feeders to prompt the acceptance of a bite); behaviours that sustain functional eating (behaviors similar to normal social activities during meals fall into this category); behaviours that extinguish functional eating (feeding episodes were extinguished when they were begun and aborted before food reached the mouth; for example, this occurred when the feeder was interrupted or distracted in the middle of the episode).
Kayser-Jones and Schell (1997a)	Ineffective mealtime strategies: Labelling resident and lack of assistance and supervision at mealtime; Providing total eating assistance and mixing food together. Effective mealtime strategies: Encouraging independence while providing supervision and assistance; creating a social mealtime environment and simplifying the process of eating.
Kayser-Jones and Schell (1997b)	The aesthetic and social dimensions of mealtimes were neglected. Residents did not receive the necessary assistance. Residents were fed forcefully.
Pierson (1999)	Findings presented under three main headings: Members’ Knowledge-“It’s just my common sense”; Spatio-temporal Accountability; Calculating and Recording Intake.
De Bellis et al. (2003)	Main themes: Approach and attitude of staff; Commitment to dementia care; Supervision and support; Role models; Family, visitors and volunteers. Major determining factors in mealtime outcomes included carer having 1. knowledge of dementia; 2. commitment to the relationship with the resident; 3. the ability to interact with the person in an appropriate way maintaining and restoring dignity and 4. taking that extra step in the care process.
Pasman et al. (2003)	Nurses used techniques to improve food intake, for example, moving a patient to a less distracting environment and softly touching the patient’s lips with a napkin to stimulate the swallowing reflex. Nurses discussed for each individual patient the right approach to maximise food intake. Nurses had different interpretations of the aversive behaviour of different patients.
Gibbs-Ward and Keller (2005)	Three themes identified: Each mealtime is a process embedded within the larger context of the care home environment; residents are central to the mealtime process through their actions and internal and external influences affect residents' actions at mealtimes.
Chang and Roberts (2008)	Three main headings: Feeding assistance provided by nursing assistants; the mealtime environment and interaction between caregivers and residents. Nursing assistants used limited strategies to deal with eating difficulty, and many did not use strategies that were effective especially when the residents refused food. Residents’ personal tastes were not considered. Nursing assistants did not communicate with residents to verify eating and food preferences or whether residents had enough to eat.
Hung and Chaudhury (2011)	Nine themes proposed in relation to personhood in dining experiences: Outpacing/relaxed pace; withholding/holding; disrespect/respect; invalidation/validation; distancing/connecting; disempowerment/empowerment; and ignoring/inclusion. The themes speak to the importance of moving away from the task-based care approaches to allow paying more careful attention to the psychosocial needs of residents.
Nell et al. (2016)	Two main themes: It’s about the individual (factors relating to the individual’s appetite (and subsequent desire for food), personal food preferences and ability to manage the eating process); It’s about the environment (factors relating to the dining environment (e.g. background music), social interactions and assistance provided by others).
Murphy et al. (2017)	One overarching theme identified: Person-centred nutritional care. Six sub-themes: Availability of food and drinks; tools, resources and equipment; relationship to others when eating and drinking; participation in activities; consistency of care and provision of information.
Palese et al. (2018)	The promotion and maintenance of eating independence for as long as possible is ensured by a set of interventions targeting three levels: (a) environmental, by ‘ritualising the mealtime experience by creating a controlled stimulated environment’; (b) social, by ‘structuring effective mealtime social interactions’ and (c) individual, by ‘individualising easting assistance’.
Driessen and Ibáñez Martín (2020)	Three ‘repertoires of difference’ presented: Providing choice (in this repertoire what is valued is being able to choose for oneself, in order to be able to eat what one feels like having in a specific moment); knowing residents (here, care workers know about singular, and relatively stable tastes and habits) and catering to identities (care workers do this by temporarily suspending their knowledge about a resident’s preferences, habits and the like. In other words, they temporarily stop knowing and tap into what is emergent in order to ‘know anew’).

**Table 3.** Thematic categories of carer–resident interaction.

Thematic category	Description	Relevant findings
Social connection	Interactions which build relationship and social connection	*Behaviors similar to normal social activities during meals (*Van Ort & Phillips, 1992, p. 252*).*
		*Creating a social mealtime environment and simplifying the process of eating* (Kayser-Jones & Schell, 1997a, p. 36)
		*The aesthetic and social dimensions of mealtimes were neglected* (Kayser-Jones & Schell, 1997b, p. 69)
		*The quality of the reciprocal relationship was significantly and positively related to the proportion of food consumed* (Amella, 1999, p. 883)
		*Withholding/holding* (Hung & Chaudhury, 2011, p. 6)
		*Distancing/connecting* (Hung & Chaudhury, 2011, p. 9)
		*Ignoring/inclusion* (Hung & Chaudhury, 2011, p. 10)
		*Structuring effective social interactions ensuring mealtime as a pleasant social experience* (Palese et al., 2019, p. 5)

Tailored care	Interactions which are tailored to the individual	*Providing adequate time in which to feed* (Suski & Nielsen, 1989, p. 1770)
		*Labelling resident and lack of assistance and supervision at mealtime* (Kayser-Jones & Schell, 1997a, p. 35)
		*Residents did not receive the necessary assistance* (Kayser-Jones & Schell, 1997b, p. 70)
		*Approach and attitude of staff* (De Bellis, 2003, p. 7)
		*Assessing the situation and reacting accordingly (*Pierson, 1999, p. 128*)*
		*Personal tastes were not considered (*Chang & Roberts, 2008, p. 238*)*
		*Outpacing/relaxed pace* (Hung & Chaudhury, 2011, pp. 5, 6)
		*Invalidation/validation* (Hung & Chaudhury, 2011, pp. 8, 9)
		*Person-centred nutritional care* (Murphy et al., 2017, p. 4)
		*Findings suggest that the use of each handfeeding technique should be considered within context of the residents’ functional ability, energy level, and individual preferences* (Batchelor-Murphy et al., 2017, p. e92)
		*It’s about the individual* (Nell et al., 2016, p. E2)
		*Individualised eating assistance* (Palese et al., 2018, p. 6)
		*Knowing residents* (Driessen & Ibáñez Martín, 2020, p. 252)
		*Catering to identities* (Driessen & Ibáñez Martín, 2020, p. 254)
Empowering the resident	Interactions which promote autonomy and independence	*The consistent use of prompting and reinforcement maximizes ADL functional status* (Coyne & Hoskins, 1997, p. 287)
		*Totally feeding the resident and mixing food together* (Kayser-Jones & Schell, 1997a, pp. 35)
		*Encouraging independence while providing supervision and assistance* (Kayser-Jones & Schell, 1997a, pp. 36, 37)
		*Wanted Control, the willingness to let another control one’s behavior, was positively correlated to the criterion [the proportion of food consumed]* (Amella, 1999, p. 883)
		*The instrument of choice for feeding was always a spoon* (Pierson, 1999, p. 130)
		*Disrespect/respect* (Hung & Chaudhury, 2011, pp. 7, 8)
		*Disempowerment/empowerment* (Hung & Chaudhury, 2011, pp. 9, 10)
		*Providing choice* (Driessen & Ibáñez Martín, 2020, p. 250)
Responding to food refusal	Interactions which carefully and skilfully address the challenge of food refusal	*Using skilful feeding techniques* (Suski & Nielsen, 1989, p. 1770)
		*Visual, tactile and verbal cues were the means used by feeders to prompt the acceptance of a bite* (Van Ort and Phillips, 1992, p. 252)
		*Residents were fed forcefully* (Kayser-Jones & Schell, 1997b, p. 70)
		*Tricks and techniques* (Pasman et al., 2003, p. 307)
		*To stop or to continue feeding* (Pasman et al., 2003, p. 307)
		*Many did not use strategies that were effective especially when the residents refused food* (Chang & Roberts, 2008, p. 238)

#### Social connection

Social connection refers to interactions which build relationship between carer and resident and which facilitate social connection at mealtimes. This sense of relationship and social connection featured in findings from six studies. Two studies identified the importance of carer–resident relationship (Amella, 1999; Hung & Chaudhury, 2011). Four studies found that social interactions were key at mealtimes (Kayser-Jones & Schell, 1997a, 1997b; Palese et al., 2018; Van Ort & Phillips, 1992).

#### Tailored care

Tailored care denotes interactions which are tailored to the individual. The idea of tailored care was represented in findings from 12 studies. Some studies emphasised tailoring the amount of direct assistance to suit the resident (Batchelor-Murphy et al., 2017; Kayser-Jones & Schell, 1997a, 1997b; Palese et al., 2018; Pierson et al., 1999). Other studies highlighted the importance of knowing residents’ preferences (Driessen & Ibáñez Martín, 2020; Murphy et al., 2017; Nell et al., 2016). Focusing on the individual resident – rather than the task – was identified in other studies (De Bellis, 2003; Hung & Chaudhury, 2011).

#### Empowering the resident

Empowering the resident is about interactions which promote the resident’s autonomy and independence. Empowerment in this way was represented in the findings of several studies. Encouragement of independent eating was advocated (Hung & Chaudhury, 2011; Kayser-Jones & Schell, 1997a). So too was ceding control to the resident in mealtime interactions (Amella, 1999; Batchelor-Murphy et al., 2017; Hung & Chaudhury, 2011). Provision of choice was a key theme in Driessen and Ibáñez Martín (2020). Pierson (1999) highlighted the problem of carers making decisions on residents’ behalf.

#### Responding to food refusal

Responding to food refusal is concerned with interactions which carefully and skilfully address the challenge of food refusal. Some studies promoted the use of skills and techniques in dealing with this challenge (Pasman et al., 2003; Suski & Nielsen, 1989; Van Ort & Phillips, 1992). The balance of encouraging oral intake without using force was also highlighted in studies (Kayser-Jones & Schell, 1997b; Pasman et al., 2003).

Engström and Hammar (2012) was included in the synthesis but did not contribute to the thematic categories. This study reported contradictory findings for the effects of carers humming during mealtimes. For one participant, oral intake was increased during the intervention, but for the other participant, it was reduced.

## Discussion

This systematic review has investigated the published evidence on mealtime care for people with dementia living in care homes. It has taken an intentionally broad, inclusive approach to study selection, and as a consequence, the included studies are heterogeneous in design. The review has focused on ways in which care staff interact with residents whilst providing mealtime care. It has identified four thematic categories of interaction across the studies: *Social connection*, *Tailored care*, *Empowering the resident* and *Responding to food refusal*.

These findings may be informative for practitioners, researchers and policy makers seeking to optimise mealtime care for people with dementia living in care homes. Firstly, the findings point to social connection as an important part of mealtime care. Care home staff are able to foster social connection at mealtimes, not only through their own interactions *with* residents but also by facilitating interactions between residents. For some staff, this may be a very natural undertaking; for others, it may have to be more intentional. Interventions promoting social connection at mealtimes should be considered (Watkins et al., 2019), but these also need to take account of residents’ individual characteristics (Cherry et al., 2008). Secondly, the findings indicate that mealtime care should be tailored to the resident. This means knowing the resident’s needs and preferences and prioritising them during mealtimes – but also being receptive to the idea that they may change (Driessen & Ibáñez Martín, 2020). Thirdly, the findings say that good mealtime care helps residents to be empowered. This can happen when residents are given choice at mealtimes (e.g. whether this is choice of what to eat, where to eat and when to eat). It can also happen when care home staff allow residents to be in control at mealtimes – for example, to eat without assistance, even if this takes longer. Complexities may arise from this, particularly when a resident’s choice is perceived as unwise. An understanding of the Mental Capacity Act is likely important in this context (Manthorpe and Samsi, 2016a). Fourthly, the findings suggest that responding carefully and skilfully to food refusal is a significant element of mealtime care. Skilled care home staff may be able to respond in a way that encourages (but does not coerce) a resident to eat more. Further work is needed to find ways to articulate and delineate this skill so that it can be replicated (Liu et al., 2020).

In reporting review findings, it is important to consider strength of evidence, the context of other literature and review methods (Moher et al., 2009).

### Strength of evidence

The strength of evidence for these thematic categories was varied. The category *Social connection* was supported by six studies. Some of these had significant shortcomings in their design or reporting. For example, the qualitative studies by De Bellis et al., (2003) and Kayser-Jones and Schell (1997a, 1997b) provided only brief or minimal illustrations from data to show the basis of their conclusions. By contrast, both Hung and Chaudhury (2011) and Palese et al. (2018) used plentiful direct quotes and data extracts to support their themes.

The concept of *Tailored care* was supported by 12 studies, easily the most of all the categories. Again, however, the quality of evidence was mixed. There was variation, for example, in the reliability of outcome measures used in the quantitative studies. Some measures of oral intake were precise and objective (Batchelor-Murphy et al., 2017), while others had greater risk of unreliability (Suski and Nielsen, 1989). There was also variation in the extent to which qualitative studies reported a congruent philosophical perspective and research methodology. Some studies clearly articulated a link between the two (De Bellis et al., 2003; Hung & Chaudhury, 2011; Pierson, 1999), while others did not (Chang & Roberts, 2008; Driessen & Ibáñez Martín, 2020; Kayser-Jones & Schell, 1997a, 1997b; Nell et al., 2016; Murphy et al., 2017; Palese et al., 2018).

Evidence for *Empowering the resident*, provided by six of the included studies, was also varied in quality. One quantitative study (Amella, 1999) met all criteria except identification of confounding factors. The other (Coyne & Hoskins, 1997) missed three criteria, partly due to lack of clarity in reporting. The qualitative studies (Driessen & Ibáñez Martín, 2020; Hung & Chaudhury, 2011; Kayser-Jones & Schell, 1997a; Pierson, 1999) were each marked down on more than one criterion – for example, none addressed influence of the researcher on the research and vice versa.

*Responding to food refusal* was supported by the lowest number of studies: five. Several of these scored poorly in quality appraisal. Two achieved half of the available criteria. Kayser-Jones and Schell (1997b), a qualitative study, was unclear on philosophical perspective, data analysis and ethical approval – and did not adequately represent participants’ voices. Suski and Nielsen (1989), a quantitative study, was unclear on how the condition was measured and on how to deal with confounding factors – and did not measure outcomes in a reliable way. A third study – Chang and Roberts (2008) – achieved only one criterion, pertaining to the connection between data and conclusions. For six other criteria, this study was rated Unclear.

### Comparison to other literature

The thematic categories generated by this review are for the most part echoed in other related literature – for example, broader mealtime care literature and dementia care literature. A number of recent studies have pointed to the importance of social interactions at mealtimes in long-term care (Chaudhury et al., 2017; Keller et al., 2018; Watkins et al., 2017). Also, current guidance has emphasised the value of social relationships and interactions more broadly for people living with dementia (Alzheimer’s Society, 2019; National Institute for Health and Care Excellence (UK), 2018). In dementia care and more widely, relationships and interactions are considered central to quality care (see, e.g. relationship-centred care (Nolan et al., 2004; Tresolini et al., 1994)).

Similarly, the idea of tailoring care to the individual resident is prevalent both in broader mealtime care literature (Reimer & Keller, 2009; Slaughter et al., 2020; Wu et al., 2018) and in dementia care literature (Fazio et al, 2018; Manthorpe and Samsi, 2016b). This concept also features prominently in guidance and regulation in this area (Care Quality Commission, 2014; Social Care Institute for Excellence, 2020).

The importance of empowering the resident is a common theme in previous work on general mealtime care. Several studies have emphasised the need for interventions which maximise independence and autonomy at mealtimes (Iuglio et al., 2018; Mann et al., 2019; Palese et al., 2019; Reimer & Keller, 2009). The topic also features in studies which explore dementia care more broadly (Boumans et al., 2019; McCormack, 2001) and in various published guidelines for dementia care (Alzheimer’s Association, 2009; Irish Nutrition and Dietetics Institute, 2016).

The way that carers respond to food refusal is less prevalent than the other categories in recent studies on mealtime care (although it is found in some older literature, particularly from Scandinavia (see Athlin & Norberg, 1987; Jansson et al., 1995) and in published guidance (Caroline Walker Trust, 1998)). At a broader level, the idea of refusal of/resistance to a care act of some kind – and the way in which staff respond to this challenge – is well documented in the dementia care literature. There are examples in the context of medication (Haskins & Wick, 2017; Young & Unger, 2016), oral hygiene (Jablonski, et al., 2011) and general care (Konno, et al., 2012; Mahoney et al., 1999). It is not clear why there is a relative absence of this theme in mealtime care literature. Perhaps it is taken for granted (and therefore sometimes unstated) that mealtime care includes response to food refusal. New research in this area may be beneficial, to better understand the challenges of food refusal, and optimal ways to respond.

### Strengths and limitations of the review

As well as considering the strength of evidence provided by included studies – and the relationship between these studies and other literature – it is important to evaluate the robustness of methods used in the review.

The review has included heterogeneous study designs. This decision was taken in order to maximise findings and the ability of those findings to inform policy and practice (Harden, 2010); an appropriate strategy to address the complexity of healthcare research questions (Bressan et al., 2017). By synthesising data from a variety of sources, the review has been able to identify broad categories of the carer–resident interaction in mealtime care. It does not, however, provide an estimate of the effectiveness of a current intervention (or type of intervention). Instead, it is intended to identify relevant principles which may help with intervention development and thus improve practice.

In keeping with this inclusive approach, the decision was taken to accept for synthesis all studies meeting the eligibility criteria for the review; that is, there was no cut-off score for quality. Instead, quality assessment was used to gain an understanding of the strength of the evidence and taken into account during the process of synthesis (Centre for Reviews and Dissemination, 2008, p. 227). There is a range of quality within the groups of studies supporting each thematic category in the synthesis, and for each category, the methodological strengths and weaknesses are reported narratively. It is therefore possible to draw only provisional conclusions about the thematic categories, and further robust evidence is needed because of the mixed quality of included studies.

As well as taking into account methodological quality as assessed by the JBI critical appraisal tools, it is worth noting studies with findings that were problematic in other ways. Engström and Hammar (2012), as previously mentioned, reported contradictory findings for their intervention. Van Ort and Phillips (1992), in attempting to develop categories of caregiver and resident behaviour, found ‘mutually exclusive categories were difficult to distinguish’ (p. 253). Pierson (1999) sought to describe the work of carers providing mealtime care but decided this an almost impossible task since it comprises embodied practices and unspoken knowledge. Chang and Roberts (2008) organised their findings under broad descriptive headings rather than discrete themes, which meant the findings were not easily amenable to the synthesis process. Coyne and Hoskins (1997) trialled their mealtime care intervention in a somewhat different way to other studies: it was intended to increase independence of residents at subsequent mealtimes, not the current mealtime.

A qualitative approach has been used in the review synthesis. Following Popay et al. (2006), it was necessary to use a common rubric for synthesis of quantitative and qualitative data, and in this case, a textual summary of results was produced for each study. There is the potential for reviewer bias in this method, but this was mitigated by using only verbatim descriptions of explicit study findings and themes whilst developing the synthesis and through regular discussion of emerging thematic categories by the review team.

The decision was made to specify people with dementia as the population of interest, rather than care home residents more generally. It is possible therefore that some studies have been excluded with useful findings on the broader topic of mealtimes in care homes. However, the particular nature of the challenges faced by people with dementia at mealtimes – and their carers – has motivated the authors to focus their research on this population.

Finally, the review has focused on carer–resident interaction at mealtimes. This focus has allowed for detailed analysis of this aspect of mealtime care as it is reported in the literature. At the same time, it must be acknowledged that there are other, organisational, factors which may impact on the way people with dementia experience mealtimes – for example, physical environment, food service and menu provision. These factors are not explored here because they have been covered in previous reviews (Abdelhamid et al., 2016; Bunn et al., 2016; Herke et al., 2018; Jackson et al., 2011; Liu et al., 2015). Nevertheless, it would be essential to consider such organisational factors in future intervention studies on this topic. Carer–resident interactions are, after all, influenced by care home environment, staffing ratios, company policies, staff training and many other things outside the immediate control of the care staff themselves. Complex interventions literature is a useful reference point here, to help researchers take account of systems and context (see O'Cathain et al., 2019; Hawe et al., 2009; May & Finch, 2009).

## Conclusion

This evidence synthesis has brought together a diverse body of data on the topic of mealtime care for people with dementia who live in care homes. It has focused on carer–resident interaction at mealtimes. Notwithstanding the aforementioned limitations, the findings indicate that good mealtime care may involve interactions which facilitate social connection, which are tailored to individual residents, which empower residents to promote autonomy and independence and which carefully and skilfully respond to the challenge of food refusal. Given the variable quality of evidence, it is not possible to make definitive practice recommendations here. Nevertheless, each of the identified thematic categories has echoes in related literature and provides promising directions for future research. They merit further consideration as new interventions are developed to improve mealtime care for this population.

## Supplemental Material

sj-pdf-1-dem-10.1177_14713012211002041 – Supplemental Material for How do we provide good mealtime care for people with dementia living in care homes? A systematic review of carer–resident interactions Click here for additional data file. Supplemental Material, sj-pdf-1-dem-10.1177_14713012211002041 for How do we provide good mealtime care for people with dementia living in care homes? A systematic review of carer–resident interactions by James Faraday, Clare Abley, Fiona Beyer, Catherine Exley, Paula Moynihan and Joanne M Patterson in Dementia

## References

[bibr1-14713012211002041] Abbott R. A. Whear R. Thompson-Coon J. Ukoumunne O. C. Rogers M. Bethel A. Hemsley A. Stein K. (2013). Effectiveness of mealtime interventions on nutritional outcomes for the elderly living in residential care: A systematic review and meta-analysis. Ageing Research Reviews, 12(4), 967-981. 23811415 10.1016/j.arr.2013.06.002

[bibr2-14713012211002041] Abdelhamid A. Bunn D. Copley M. Cowap V. Dickinson A. Gray L. Howe A. Killett A. Lee J. Li F. Poland F. Potter J. Richardson K. Smithard D. Fox C. Hooper L. (2016). Effectiveness of interventions to directly support food and drink intake in people with dementia: Systematic review and meta-analysis. BMC Geriatrics, 16(1), 26. 26801619 10.1186/s12877-016-0196-3 PMC4722767

[bibr3-14713012211002041] Alagiakrishnan K. Bhanji R. A. Kurian M. (2013). Evaluation and management of oropharyngeal dysphagia in different types of dementia: A systematic review. Archives of gerontology and geriatrics, 56(1), 1-9. 22608838 10.1016/j.archger.2012.04.011

[bibr91-14713012211002041] Alzheimer’s Association. (2009). Dementia care practice recommendations for assisted living residences and nursing homes. Retrieved from: https://alz.org/media/Documents/dementia-care-practice-recommend-assist-living-1-2-b.pdf

[bibr4-14713012211002041] Alzheimer’s Society. (2013). Low expectations: Attitudes on choice, care and community for people with dementia in care homes*.*

[bibr5-14713012211002041] Alzheimer’s Society. (2014). Dementia UK update*.*

[bibr6-14713012211002041] Alzheimer’s Society. (2019). Person-centred care*.* Retrieved from: https://www.alzheimers.org.uk/about-dementia/treatments/person-centred-care.

[bibr7-14713012211002041] Amella E. J. (1999). Factors influencing the proportion of food consumed by nursing home residents with dementia. Journal of the American Geriatrics Society, 47(7), 879-885. 10404936 10.1111/j.1532-5415.1999.tb03849.x

[bibr8-14713012211002041] Amella E. J. (2002). Resistance at mealtimes for persons with dementia. Journal of Nutrition Health and Aging, 6(2), 117-122. 12166364

[bibr9-14713012211002041] Aromataris E. Munn Z. (2017). Joanna Briggs Institute reviewer’s manual*.* Retrieved from: https://wiki.joannabriggs.org/display/MANUAL/JBI+Reviewer%27s+Manual. 10.1097/XEB.0000000000000155 30256247

[bibr10-14713012211002041] Aselage M. B. Amella E. J. Watson R. (2011). State of the science: Alleviating mealtime difficulties in nursing home residents with dementia. Nursing Outlook, 59, 210-214. 21757077 10.1016/j.outlook.2011.05.009

[bibr11-14713012211002041] Athlin E. Norberg A. (1987). Caregivers' attitudes to and interpretations of the behaviour of severely demented patients during feeding in a patient assignment care system. International Journal of Nursing Studies, 24(2), 145-153. 3647000 10.1016/0020-7489(87)90056-3

[bibr12-14713012211002041] Australian Institute of Health and Welfare. (2012). Residential aged care in Australia 2010–11: A statistical overview*.*

[bibr13-14713012211002041] Batchelor-Murphy M. K. McConnell E. S. Amella E. J. Anderson R. A. Bales C. W. Silva S. Barnes A. Beck C. Colon-Emeric C. S. (2017). Experimental comparison of efficacy for three handfeeding techniques in dementia. Journal of the American Geriatrics Society, 65(4), e89-e94. 28165618 10.1111/jgs.14728 PMC5397360

[bibr14-14713012211002041] Boumans J. Van Boekel L. C. Baan C. A. Luijkx K. G. (2019). How can autonomy be maintained and informal care improved for people with dementia living in residential care facilities: A systematic literature review. The Gerontologist, 59(6), e709-e730. 30239712 10.1093/geront/gny096 PMC6858830

[bibr15-14713012211002041] Bressan V. Bagnasco A. Aleo G. Timmins F. Barisone M. Bianchi M. Pellegrini R. Sasso L. (2017). Mixed-methods research in nursing - A critical review. Journal of Clinical Nursing, 26(19-20), 2878-2890. 27805742 10.1111/jocn.13631

[bibr16-14713012211002041] Brush J. A. Calkins M. P. (2008). Environmental interventions and dementia: Enhancing mealtimes in group dining rooms. The ASHA Leader, 13(8), 24-25.

[bibr17-14713012211002041] Bunn D. K. Abdelhamid A. Copley M. Cowap V. Dickinson A. Howe A. Killett A. Poland F. Potter JF. Richardson K. Smithard D. Fox C. Hooper L. (2016). Effectiveness of interventions to indirectly support food and drink intake in people with dementia: Eating and drinking well IN dementiA (EDWINA) systematic review. BMC Geriatrics, 16(1), 89. 27142469 10.1186/s12877-016-0256-8 PMC4855348

[bibr18-14713012211002041] Burges Watson D. L. Lewis S. Bryant V. Patterson J. Kelly C. Edwards-Stuart R. Murtagh MJ Deary V. (2018). Altered eating: A definition and framework for assessment and intervention. BMC Nutrition, 4(1), 14. 32153878 10.1186/s40795-018-0221-3 PMC7050903

[bibr19-14713012211002041] Burnard P. Gill P. Stewart K. Treasure E. Chadwick B. (2008). Analysing and presenting qualitative data. British Dental Journal, 204(8), 429-432. 18438371 10.1038/sj.bdj.2008.292

[bibr20-14713012211002041] Care Quality Commission (2014). Regulation 9: Person-centred care. Retrieved from: https://www.cqc.org.uk/guidance-providers/regulations-enforcement/regulation-9-person-centred-care.

[bibr21-14713012211002041] Caroline Walker Trust (1998). Eating well for older people with dementia*.*

[bibr22-14713012211002041] Centre for Reviews and Dissemination (2008). Systematic reviews: CRD’s guidance for undertaking reviews in healthcare.

[bibr23-14713012211002041] Chang C.-C. Roberts B. L. (2008). Cultural perspectives in feeding difficulty in Taiwanese elderly with dementia. Journal of Nursing Scholarship, 40(3), 235-240. 18840206 10.1111/j.1547-5069.2008.00231.x

[bibr24-14713012211002041] Chaudhury H. Keller H. Pfisterer K. Hung L. (2017). Development of a physical environmental observational tool for dining environments in long-term care settings. The Gerontologist, 57(6), e95-e101. 28329819 10.1093/geront/gnw261

[bibr25-14713012211002041] Cherry B. Carpenter K. Waters C. Hawkins W. W. McGrew P. Satterwhite L. J. Stepien J. Ruppelt W. Herring K. (2008). Social compatibility as a consideration in caring for nursing home residents with dementia. American Journal of Alzheimer's Disease and Other Dementias, 23(5), 430-438. 10.1177/1533317508326046 PMC10846234 18955722

[bibr26-14713012211002041] Coyne M. L. Hoskins L. (1997). Improving eating behaviors in dementia using behavioral strategies. Clinical Nursing Research, 6(3), 275-290. 9281930 10.1177/105477389700600307

[bibr27-14713012211002041] De Bellis A. Willick C. Mitchell P. Roder-Allen G. (2003). Food for thought: Residents with dementia who require assistance with eating and drinking. Geriaction, 21(3), 5-10.

[bibr28-14713012211002041] Department of Health (UK) (2015). Prime Minister’s challenge on dementia 2020.

[bibr29-14713012211002041] Driessen A. Ibáñez Martín R. (2020). Attending to difference: enacting individuals in food provision for residents with dementia. Sociology of Health & Illness, 42(2), 247-261. 31705562 10.1111/1467-9566.13004

[bibr30-14713012211002041] Engström G. Hammar L. M. (2012). Humming as a potential tool for facilitating feeding situations between persons with dementia and their caregiver: A single case study. Music and Medicine, 4(4), 231-236.

[bibr31-14713012211002041] Evans G. Smith A. Morrow K. (2009). Compromised swallowing: A practical guide to nutrition, hydration and medication in advanced dementia*.* Palliative Care in Dementia Group.

[bibr32-14713012211002041] Fazio S. Pace D. Flinner J. Kallmyer B. (2018). The fundamentals of person‐centered care for individuals with dementia. The Gerontologist, 58(Suppl. 1), S10-S19. 29361064 10.1093/geront/gnx122

[bibr33-14713012211002041] Fetherstonhaugh D. Haesler E. Bauer M. (2019). Promoting mealtime function in people with dementia: A systematic review of studies undertaken in residential aged care. International Journal of Nursing Studies, 96, 99-118. 31060734 10.1016/j.ijnurstu.2019.04.005

[bibr34-14713012211002041] Gibbs-Ward A. J. Keller H. H. (2005). Mealtimes as active processes in long-term care facilities. Canadian Journal of Dietetic Practice and Research, 66(1), 5-11. 15780150 10.3148/66.1.2005.5

[bibr35-14713012211002041] Glaser B. G. (1965). The constant comparative method of qualitative analysis. Social Problems, 12(4), 436-445.

[bibr36-14713012211002041] Hanson L. C. Ersek M. Lin F. C. Carey T. S. (2013). Outcomes of feeding problems in advanced dementia in a nursing home population. Journal of the American Geriatrics Society, 61(10), 1692-1697. 24083403 10.1111/jgs.12448 PMC4385385

[bibr37-14713012211002041] Harden A. (2010). Mixed-methods systematic reviews: Integrating quantitative and qualitative findings. Focus. 25, 1-8

[bibr38-14713012211002041] Harris-Kojetin L. Sengupta M. Park-Lee E. Valverde R. Caffrey C. Rome V. Lendon J. (2016). Long-term care providers and services users in the United States: Data from the national study of long-term care providers, 2013-2014. Vital Health Statistics, 3(38), 1-105. 27023287

[bibr39-14713012211002041] Haskins D. R. Wick J. Y. (2017). Medication refusal: Resident rights, administration dilemma. The Consultant Pharmacist, 32(12), 728-736. 29467065 10.4140/TCP.n.2017.728

[bibr40-14713012211002041] Hawe P. Shiell A. Riley T. (2009). Theorising interventions as events in systems. American Journal of Community Psychology, 43(3-4), 267-276. 19390961 10.1007/s10464-009-9229-9

[bibr41-14713012211002041] Health Education England. (2015). The care certificate workbook, standard 8: Fluids and nutrition.

[bibr42-14713012211002041] Herke M. Fink A. Langer G. Wustmann T. Watzke S. Hanff A. M. Burckhardt M. (2018). Environmental and behavioural modifications for improving food and fluid intake in people with dementia. Cochrane Database of Systematic Reviews, 7, CD011542. 10.1002/14651858.CD011542.pub2 PMC6513567 30021248

[bibr43-14713012211002041] Hong Q. N. Pluye P. Bujold M. Wassef M. (2017). Convergent and sequential synthesis designs: Implications for conducting and reporting systematic reviews of qualitative and quantitative evidence. Systematic Reviews, 6(1), 61. 28335799 10.1186/s13643-017-0454-2 PMC5364694

[bibr44-14713012211002041] Hung L. Chaudhury H. (2011). Exploring personhood in dining experiences of residents with dementia in long-term care facilities. Journal of Aging Studies, 25(1), 1-12.

[bibr45-14713012211002041] Ikeda M. Brown J. Holland A. J. Fukuhara R. Hodges J. R. (2002). Changes in appetite, food preference, and eating habits in frontotemporal dementia and Alzheimer’s disease. Journal of Neurology, Neurosurgery, and Psychiatry, 73(4), 371-376. 10.1136/jnnp.73.4.371 PMC1738075 12235302

[bibr46-14713012211002041] Iuglio S. Keller H. Chaudhury H. Slaughter S. E. Lengyel C. Morrison J. Boscart V Carrier N. (2018). Construct validity of the dining environment audit protocol: A secondary data analysis of the making most of mealtimes (M3) study. BMC Geriatrics, 18(1), 20. 29357821 10.1186/s12877-018-0708-4 PMC5778623

[bibr92-14713012211002041] Irish Nutrition Dietetics Institute. (2016). Nutrition and dementia. Retrieved from: https://www.indi.ie/images/Dementia_Booklet__.pdf

[bibr47-14713012211002041] Jablonski R. A. Kolanowski A. Therrien B. Mahoney E. K. Kassab C. Leslie D. L. (2011). Reducing care-resistant behaviors during oral hygiene in persons with dementia. BMC Oral Health, 11(1), 30. 22100010 10.1186/1472-6831-11-30 PMC3231974

[bibr48-14713012211002041] Jackson J. Currie K. Graham C. Robb Y. (2011). The effectiveness of interventions to reduce undernutrition and promote eating in older adults with dementia: A systematic review. JBI Database of Systematic Reviews and Implementation Reports, 9(37), 1509-1550. 10.11124/01938924-201109370-00001 27819926

[bibr49-14713012211002041] Jansson L. Norberg A. Sandman P.-O. Åström G. (1995). When the severely ill elderly patient refuses food: Ethical reasoning among nurses. International Journal of Nursing Studies, 32(1), 68-78. 7730007 10.1016/0020-7489(94)00024-e

[bibr50-14713012211002041] Kai K. Hashimoto M. Amano K. Tanaka H. Fukuhara R. Ikeda M. (2015). Relationship between eating disturbance and dementia severity in patients with Alzheimer’s disease. PloS one, 10(8), e0133666. 26266531 10.1371/journal.pone.0133666 PMC4534398

[bibr51-14713012211002041] Kayser-Jones J. Schell E. (1997a). The Mealtime Experience of a cognitively impaired elder: Ineffective and effective strategies. Journal of Gerontological Nursing, 23(7), 33-39. 10.3928/0098-9134-19970701-11 9287604

[bibr52-14713012211002041] Kayser-Jones J. Schell E. (1997b). Staffing and the mealtime experience of nursing home residents on a special care unit. American Journal of Alzheimer’s Disease, 12(2), 67-72.

[bibr53-14713012211002041] Keller H. H. (2016). Improving food intake in persons living with dementia. Annals of the New York Academy of Sciences, 1367(1), 3-11. 26773443 10.1111/nyas.12997

[bibr54-14713012211002041] Keller H. H. Chaudhury H. Pfisterer K. J. Slaughter S. E. (2018). Development and inter-rater reliability of the mealtime scan for long-term care. The Gerontologist, 58(3), e160-e167. 28329849 10.1093/geront/gnw264 PMC5946921

[bibr55-14713012211002041] Kitwood T. (1997). Dementia reconsidered: the person comes first*.* Open University Press.

[bibr56-14713012211002041] Konno R. Kang H. S. Makimoto K. (2012). The best evidence for minimizing resistance-to-care during assisted personal care for older adults with dementia in nursing homes: a systematic review. JBI Library of Systematic Reviews, 10(58), 4622-4632. 27820527 10.11124/jbisrir-2012-431

[bibr57-14713012211002041] Liu W. Galik E. Boltz M. Nahm E.-S. Resnick B. (2015). Optimizing eating performance for older adults with dementia living in long-term care: A systematic review. Worldviews on Evidence-Based Nursing, 12(4), 228-235. 26122316 10.1111/wvn.12100

[bibr58-14713012211002041] Liu W. Tripp-Rei mer T. Williams K. Shaw C. (2020). Facilitators and barriers to optimizing eating performance among cognitively impaired older adults: A qualitative study of nursing assistants’ perspectives. Dementia, 19(6), 2090-2113. 30482090 10.1177/1471301218815053

[bibr59-14713012211002041] Mahoney E. K. Hurley A. C. Volicer L. Bell M. Gianotis P. Hartshorn M. Lane P. Lesperance R. MacDonald S. Novakoff L. Rheaume Y. Timms R. Warden V. (1999). Development and testing of the resistiveness to care scale. Research in Nursing & Health, 22(1), 27-38. 9928961 10.1002/(sici)1098-240x(199902)22:1<27::aid-nur4>3.0.co;2-t

[bibr60-14713012211002041] Mann K. Lengyel C. O. Slaughter S. E. Carrier N. Keller H. (2019). Resident and staff mealtime actions and energy intake of long-term care residents with cognitive impairment: Analysis of the making the most of mealtimes study. Journal of Gerontological Nursing, 45(8), 32-42. 31355897 10.3928/00989134-20190709-04

[bibr61-14713012211002041] Manthorpe J. Samsi K. (2016a). Care homes and the mental capacity act 2005: Changes in understanding and practice over time. Dementia, 15(4), 858-871. 25015949 10.1177/1471301214542623

[bibr62-14713012211002041] Manthorpe J. Samsi K. (2016b). Person-centered dementia care: Current perspectives. Clinical Interventions in Aging, 11, 1733. 27932869 10.2147/CIA.S104618 PMC5135058

[bibr63-14713012211002041] May C. Finch T. (2009). Implementing, embedding, and integrating practices: An outline of normalization process theory. Sociology, 43(3), 535-554.

[bibr64-14713012211002041] McCormack B. (2001). Autonomy and the relationship between nurses and older people. Ageing and Society, 21, 417-446.

[bibr65-14713012211002041] Moher D. Liberati A. Tetzlaff J. Altman D. G., & PRISMA Group. (2009). Preferred reporting items for systematic reviews and meta-analyses: The PRISMA statement. PLOS Medicine, 6(7), e1000097. 19621072 10.1371/journal.pmed.1000097 PMC2707599

[bibr66-14713012211002041] Murphy J. L. Holmes J. Brooks C. (2017). Nutrition and dementia care: Developing an evidence-based model for nutritional care in nursing homes. BMC Geriatrics, 17(1), 55. 28196475 10.1186/s12877-017-0443-2 PMC5309970

[bibr67-14713012211002041] National Institute for Health and Care Excellence (UK). (2018). Dementia: Assessment, management and support for people living with dementia and their carers. NICE guideline [NG97]. Retrieved from https://www.nice.org.uk/guidance/ng97. 30011160

[bibr68-14713012211002041] Nell D. Neville S. Bellew R. O'Leary C. Beck K. L. (2016). Factors affecting optimal nutrition and hydration for people living in specialised dementia care units: A qualitative study of staff caregivers' perceptions. Australasian Journal on Ageing, 35(4), E1-E6. 10.1111/ajag.12307 26969881

[bibr69-14713012211002041] Ney D. M. Weiss J. M. Kind A. J. Robbins J. (2009). Senescent swallowing: impact, strategies, and interventions. Nutrition in Clinical Practice, 24(3), 395-413. 19483069 10.1177/0884533609332005 PMC2832792

[bibr70-14713012211002041] Nolan M. R. Davies S. Brown J. Keady J. Nolan J. (2004). Beyond person-centred care: A new vision for gerontological nursing. Journal of Clinical Nursing, 13(3a), 45-53. 15028039 10.1111/j.1365-2702.2004.00926.x

[bibr71-14713012211002041] O’Cathain A. Croot L. Duncan E. Rousseau N. Sworn K. Turner K. M. Yardley L. Hoddinott P. (2019). Guidance on how to develop complex interventions to improve health and healthcare. BMJ Open, 9(8), e029954. 10.1136/bmjopen-2019-029954 PMC6701588 31420394

[bibr72-14713012211002041] Palese A. Bressan V. Kasa T. Meri M. Hayter M. Watson R. (2018). Interventions maintaining eating independence in nursing home residents: A multicentre qualitative study. BMC Geriatrics, 18(1), 292. 30482168 10.1186/s12877-018-0985-y PMC6258290

[bibr73-14713012211002041] Palese A. Grassetti L. Bressan V. Decaro A. Kasa T. Longobardi M. Hayter M. Watson R. (2019). A path analysis on the direct and indirect effects of the unit environment on eating dependence among cognitively impaired nursing home residents. BMC Health Services Research, 19(1), 775. 31666063 10.1186/s12913-019-4667-z PMC6822399

[bibr74-14713012211002041] Pasman H. R. The B. A. Onwuteaka-Philipsen B. D. Van der Wal G. Ribbe M. W. (2003). Feeding nursing home patients with severe dementia: A qualitative study. Journal of Advanced Nursing, 42, 304-311. 12680975 10.1046/j.1365-2648.2003.02620.x

[bibr75-14713012211002041] Pierson C. A. (1999). Ethnomethodologic analysis of accounts of feeding demented residents in long-term care. Journal of Nursing Scholarship, 31(2), 127-131. 10.1111/j.1547-5069.1999.tb00447.x 10380387

[bibr76-14713012211002041] Popay J. Roberts H. M. Sowden A. Petticrew M. Arai L. Rodgers M. Britten N. (2006). Guidance on the conduct of narrative synthesis in systematic reviews*.* Institute for Health Research

[bibr77-14713012211002041] Prince M. Albanese E. Guerchet M. Prina M. (2014). Nutrition and dementia A review of available research*.* Alzheimer’s Disease International.

[bibr78-14713012211002041] Reimer H. D. Keller H. H. (2009). Mealtimes in nursing homes: Striving for person-centered care. Journal of Nutrition for the Elderly, 28(4), 327-347. 21184376 10.1080/01639360903417066

[bibr79-14713012211002041] Richardson B. (2015). Nutrition to reduce hospital readmissions for older adults. Nutrition and foodservice edge. 16-19.

[bibr80-14713012211002041] Slaughter S. E. Morrison-Koechl J. M. Chaudhury H. Lengyel C. O. Carrier N. Keller H. H. (2020). The association of eating challenges with energy intake is moderated by the mealtime environment in residential care homes. International Psychogeriatrics, 32, 863-873. 31915088 10.1017/S1041610219001959

[bibr81-14713012211002041] Social Care Institute for Excellence (2015). Promoting independence at mealtimes for people with dementia*.* Retrieved from: https://www.scie.org.uk/dementia/living-with-dementia/eating-well/independence-at-mealtimes.asp.

[bibr82-14713012211002041] Social Care Institute for Excellence (2020). Evaluating personalised care: A practical guide*.*

[bibr83-14713012211002041] Suski N. S. Nielsen C. C. (1989). Factors affecting food intake of women with Alzheimer’s type dementia in long-term care. Journal of the American Dietetic Association, 89, 1770-1773. 2512336

[bibr84-14713012211002041] Torres A. Peetermans W. E. Viegi G. Blasi F. (2013). Risk factors for community-acquired pneumonia in adults in Europe: A literature review. Thorax, 68(11), 1057-1065. 24130229 10.1136/thoraxjnl-2013-204282 PMC3812874

[bibr85-14713012211002041] Tresolini C.P., & The Pew-Fetzer Task Force. (1994). Health professions education and relationships-centred care: A report of the pew-fetzer task force on advancing psychosocial education*.* Pew Health Professions Commission.

[bibr86-14713012211002041] Van Ort S. Phillips L. (1992). Feeding nursing home residents with Alzheimer's disease. Geriatric Nursing, 13(5), 249-253. 1327986 10.1016/s0197-4572(05)80413-6

[bibr87-14713012211002041] Watkins R. Goodwin V. A. Abbott R. A. Hall A. Tarrant M. (2017). Exploring residents’ experiences of mealtimes in care homes: A qualitative interview study. BMC Geriatrics, 17, 141. 28697747 10.1186/s12877-017-0540-2 PMC5506571

[bibr88-14713012211002041] Watkins R. Goodwin V. A. Abbott R. A. Tarrant M. (2019). Eating well in care homes: Testing the feasibility of a staff training programme aimed at improving social interaction and choice at mealtimes. International Journal of Older People Nursing, 14(3), e12247. 31199086 10.1111/opn.12247

[bibr89-14713012211002041] Wu S. Morrison J. M. Dunn-Ridgeway H. Vucea V. Iuglio S. Keller H. (2018). Mixed methods developmental evaluation of the CHOICE program: A relationship-centred mealtime intervention for long-term care. BMC Geriatrics, 18(1), 277. 30424725 10.1186/s12877-018-0964-3 PMC6234643

[bibr90-14713012211002041] Young J. M. Unger D. (2016). Covert administration of medication to persons with dementia: Exploring ethical dimensions. The Journal of Clinical Ethics, 27(4), 290-297. 28001136

