# Supplementary material for: How do we provide good mealtime care for people with dementia living in care homes? A systematic review of carer–resident interactions
Source: Dementia (London). 2021 Apr 7;20(8):3006–31. doi: 10.1177/14713012211002041 (PMC8679165; doi:10.1177/14713012211002041)
Supplement: sj-pdf-1-dem-10.1177_14713012211002041 – Supplemental Material for How do we provide good mealtime care for people with dementia living in care homes? A systematic review of carer–resident interactions [file sj-pdf-1-dem-10.1177_14713012211002041.pdf]

Supplemental Appendix A. MEDLINE search strategy

1. exp DEMENTIA/
2. "COGNITION DISORDERS"/ OR "COGNITIVE DYSFUNCTION"/
3. (dement*).ti,ab
4. (Alzheimer*).ti,ab
5. (1 OR 2 OR 3 OR 4)
6. DEGLUTITION/
7. "DEGLUTITION DISORDERS"/
8. exp EATING/
9. exp MEALS/
10. FOOD/
11. (eat*).ti,ab
12. (drink*).ti,ab
13. (meal*).ti,ab
14. (swallow*).ti,ab
15. (dysphagi*).ti,ab
16. (feed*).ti,ab
17. (food*).ti,ab
18. (6 OR 7 OR 8 OR 9 OR 10 OR 11 OR 12 OR 13 OR 14 OR 15 OR 16 OR 17)
19. (carer*).ti,ab
20. (caring).ti,ab
21. CAREGIVERS/
22. "PATIENT CARE"/ OR "CUSTODIAL CARE"/ OR "LONG-TERM CARE"/ OR "NURSING CARE"/
23. NURSES/
24. "NURSES' AIDES"/
25. "NURSING STAFF"/
26. (nurs*).ti,ab
27. exp "RESIDENTIAL FACILITIES"/
28. ("care home*").ti,ab
29. ("care worker*").ti,ab
30. (caregiver*).ti,ab
31. ("long-term care" OR "long term care").ti,ab
32. (19 OR 20 OR 21 OR 22 OR 23 OR 24 OR 25 OR 26 OR 27 OR 28 OR 29 OR 30 OR 32)
33. (5 AND 18 AND 33)

Supplemental Appendix B. Full-text articles excluded, with reasons for exclusion

Article	Reason for exclusion
Akerlund, B., & Norberg, A. (1985). An ethical analysis of double bind conflicts as experienced by care workers feeding severely demented patients. *International Journal of Nursing Studies, 22*, 207–216.	Not all participants in care homes
Altus, D. (2002). Using Family-Style Meals to Increase Participation and Communication in Persons with Dementia. *Journal of Gerontological Nursing, 28(9),* 47-53.	Outside direct control of care staff
Amella, E. J. & Batchelor-Aselage, M. B. (2014). Facilitating ADLs by caregivers of persons with dementia: the C3P model. *Occupational Therapy in Health Care, 28(1),* 51-61.	Not a primary study
Amella, E. J. & Lawrence, J. F. (2008). Eating and Feeding Issues in Older Adults with Dementia: Part I: Assessment. *Annals of Long-Term Care, 16(3).*	Not all participants in care homes
Amella, E. J. & Lawrence, J. F. (2008). Eating and Feeding Issues in Older Adults with Dementia: Part II: Interventions. *Annals of Long-Term Care, 16(4).*	Not all participants in care homes
Aparanji, K. P. & Dharmarajan, T.S. (2010). Pause Before a PEG: A Feeding Tube May Not Be Necessary in Every Candidate!, *Journal of the American Medical Directors Association, 11(6),* 453-456.	Outside direct control of care staff
Athlin, E. & Norberg, A. (1987). Interaction between the severely demented patient and his caregiver during feeding. A theoretical model. *Scandinavian Journal of Caring Sciences, 1(3-4),* 117–123.	Focus is assessment not care
Athlin, E. & Norberg A. (1987). Caregivers' attitudes to and interpretations of the behaviour of severely demented patients during feeding in a patient assignment care system. *International Journal of Nursing Studies, 24(2),* 145–153.	Outside direct control of care staff
Athlin, E., Norberg, A., & Asplund, K. (1990). Caregivers' perceptions and interpretations of severely demented patients during feeding in a task assignment system. *Scandinavian Journal of Caring Sciences, 4(4),* 147–156.	Not all participants in care homes
Athlin E. & Norberg A. (1998) Interaction between patients with severe dementia and their caregivers during feeding in a task-assignment versus a patient-assignment care system. *European Nurse, 3,* 215–227.	Study reported in another article
Athlin, E., Norberg, A., Asplund, K. and Jansson, L. (1989). Feeding Problems in Severely Demented Patients Seen from Task and Relationship Aspects. *Scandinavian Journal of Caring Sciences, 3(3),* 113-121.	Focus is assessment not care
Austbø Holteng, L., Frøiland, C., Corbett, A., & Testad, I. (2017). Care staff perspective on use of texture modified food in care home residents with dysphagia and dementia. *Annals Of Palliative Medicine, 6(4),* 310-318.	Outside direct control of care staff
Bäckström, Å., Norberg, A., & Norberg, B. (1987). Feeding difficulties in long-stay patients at nursing homes. Caregiver turnover and caregivers' assessments of duration and difficulty of assisted feeding and amount of food received by the patient. *International Journal of Nursing Studies 24(1),* 69–76.	Focus is assessment not care
Ball, L., Jansen, S., Desbrow, B., Morgan, K., Moyle, W., & Hughes, R. (2015). Experiences and nutrition support	Not all participants in care homes

strategies in dementia care: Lessons from family carers. *Nutrition & Dietetics, 72(1),* 22– 29.	
Barnes S., Wasielewska C., Raiswell C., & Drummond B. (2013). Exploring the mealtime experience in residential care settings for older people: an observational study. *Health and Social Care in the Community 21(4),* 442–450.	Population focus is not people with dementia
Batchelor-Murphy, M., Amella, E. J., Zapka, J., Mueller, M., & Beck, C. (2015). Feasibility of a web-based dementia feeding skills training program for nursing home staff. *Geriatric Nursing, 36(3)*, 212–218.	Outside direct control of care staff
Baur, V., & Abma, T. (2012). 'The Taste Buddies': Participation and empowerment in a residential home for older people. Ageing and Society, 32(6), 1055-1078.	Population focus is not people with dementia
Bautmans, I.; Demarteau, J., Cruts, B., Lemper, J-C., & Mets, T. (2008). Dysphagia in elderly nursing home residents with severe cognitive impairment. *Journal of Rehabilitation Medicine, 40(9),* 755-760.	Outside direct control of care staff
Beattie, E. R. A., Algase, D. L., & Song, J. (2004) Special Section—Behavioral symptoms of dementia: their measurement and intervention. Keeping wandering nursing home residents at the table: improving food intake using a behavioral communication intervention, *Aging & Mental Health, 8(2),* 109-116.	Outside direct control of care staff
Beattie, E., O'Reilly, M., Strange, E., Franklin, S., & Isenring, E. (2014). How much do residential aged care staff members know about the nutritional needs of residents? *International Journal of Older People Nursing, 9(1),* 54–64.	Outside direct control of care staff
Beel-Bates, C., Stephenson, P.L., Nochera, C.L., & Rogers, J.F. (2012). Caregiver-resident interaction with Barnard's feeding scale. *Research in Gerontological Nursing, 5(4),* 284-293.	Focus is assessment not care
Benati, G., Coppola, D., & Delvecchio, S. (2009). Staff training effect on the management of patients with dysphagia and dementia in a nursing home. *Nutritional Therapy & Metabolism, 27(2),* 95-99.	Outside direct control of care staff
Berkhout, A. M. M.., Cools, H. J. M., & Van Houwelingen, H. C. (1998). The relationship between difficulties in feeding oneself and loss of weight in nursing-home patients with dementia. *Age and Ageing, 27(5),* 637–641	Focus is assessment not care
Calkins, M. P. (2007). Exploring options along the safety-autonomy continuum. *Alzheimers Care Quarterly, 8(3),* 206.	Focus is not mealtime care activities
Caspar, S., Davis, E., Berg, K., Slaughter, S., Keller, H., & Kellett, P. (2020). Stakeholder Engagement in Practice Change: Enabling Person-Centred Mealtime Experiences in Residential Care Homes. *Canadian Journal on Aging / La Revue Canadienne Du Vieillissement*, 1-15.	Outside direct control of care staff
Chang, C.-C., & Lin, L-C. (2005). Effects of a feeding skills training programme on nursing assistants and dementia patients. *Journal of Clinical Nursing, 14,* 1185–1192.	Outside direct control of care staff
Chang, C.-C., Wykle, M. L., & Madigan, E. A. (2006). The effect of a feeding skills training program for nursing assistants who feed dementia patients in Taiwanese nursing homes. *Geriatric Nursing, 27(4),* 229–237.	Outside direct control of care staff
Chang, F.-Y., Huang, H.-C., Lin, K.-C., & Lin, L.-C. (2010). The effect of a music programme during lunchtime on	Outside direct control of care staff

the problem behaviour of the older residents with dementia at an institution in Taiwan. *Journal of Clinical Nursing, 19,* 939–948.	
Chang, E., Brownhill, S., Bidewell, J., Johnson, A., & Ratnayake, S. (2015). Focus on feeding! Evaluation of a framework for maximizing mealtime in aged care facilities. *International Journal of Nursing Practice, 21, 269– 277*.	Population focus is not people with dementia
Charras, K., & Fremontier, M. (2010). Sharing meals with institutionalized people with dementia: A natural experiment. *Journal of Gerontological Social Work, 53(5),* 436–448.	Outside direct control of care staff
Chaudhury, H., Hung, L., Rust, T., & Wu, S. (2017). Do physical environmental changes make a difference? Supporting person-centered care at mealtimes in nursing homes. *Dementia, 16(7),* 878–896.	Population focus is not people with dementia
Chen, L. L., Li, H., Lin, R., Zheng, J. H., Wei, Y. P., Li, J., . . . Chen, H. Y. (2016). Effects of a feeding intervention in patients with Alzheimer's disease and dysphagia. *Journal of Clinical Nursing, 25(5-6),* 699–707.	Outside direct control of care staff
Chiang, C. K., & Hwu, Y. J. (2018). Feeding experiences of nursing aides for residents with dysphagia. *Geriatric Nursing, 39(4),* 436- 442.	Population focus is not people with dementia
Chouinard, J., Lavigne, E. & Villeneuve, C. (1998). Weight Loss, Dysphagia, and Outcome in Advanced Dementia. *Dysphagia, 13,* 151–155.	Focus is not mealtime care activities
Cleary, S. (2009). Using environmental interventions to facilitate eating and swallowing in residents with dementia. *Canadian Nursing Home, 20(2),* 5-12.	Outside direct control of care staff
Cleary, S., Hopper, T., Forseth, M., & Van Soest, D. Using routine seating plans to improve mealtimes for residents with dementia. *Canadian Nursing Home, 19(3),* 4-10.	Outside direct control of care staff
Cluskey, M., & Kim, Y. K. (2001). Use and perceived effectiveness of strategies for enhancing food and nutrient intakes among elderly persons in long-term care. *Journal of the American Dietetic Association, 101(1),* 111-114.	Population focus is not people with dementia
Cohen, D., Post, S. G., Lo, A., Lombardo, R., & Pfeffer, B. (2020). "Music & Memory" and improved swallowing in advanced dementia. *Dementia, 19(2),* 195–204.	Outside direct control of care staff
Corcoran, M. A., & Gitlin, L. N. (1996). Managing Dementia at Home: The Role of Home Environmental Modifications. *Topics in Geriatric Rehabilitation, 12(2),* 63-69.	Not all participants in care homes
Crack, J., & Crack. G. (2007). Promoting quality care for older people in meal management: whose responsibility is it? *Australian Journal of Advanced Nursing, 25(1),* 85-89.	Population focus is not people with dementia
Davies, N., Mathew, R., Wilcock, J., Manthorpe, J., Sampson, E. L., Lamahewa, K., & Iliffe, S. (2016). A co-design process developing heuristics for practitioners providing end of life care for people with dementia. *BMC Palliative Care, 15(1),* 68.	Outside direct control of care staff
Denney, A. (1997). Quiet music. An intervention for mealtime agitation? *Journal of Gerontological Nursing, 23(7),*	Outside direct control of care staff

16-23.	
Desai, J., Winter, A., Young, K.W.H., & Greenwood, C.E. (2007). Changes in type of foodservice and dining room environment preferentially benefit institutionalized seniors with low body mass indexes. *Journal of the American Dietetic Association, 107(5),* 808-814.	Population focus is not people with dementia
Durkin, D. W., Shotwell, M. S., & Simmons, S. F. (2014). The Impact of Family Visitation on Feeding Assistance Quality in Nursing Homes. *Journal of Applied Gerontology, 33(5),* 586–602.	Outside direct control of care staff
Durnbaugh, T., Haley, B., & Roberts, S. (1996). Assessing problem feeding behaviors in mid-stage Alzheimer's disease. *Geriatric Nursing, 17(2),* 63-67.	Focus is assessment not care
Genoe, M., Keller, H., Martin, L., Dupuis, S., Reimer, H., Cassolato, C., & Edward, G. (2012). Adjusting to Mealtime Change within the Context of Dementia. *Canadian Journal on Aging / La Revue Canadienne Du Vieillissement, 31(2),* 173-194.	Not all participants in care homes
Gilmore-Bykovskyi, A. L. (2015). Caregiver person-centeredness and behavioral symptoms during mealtime interactions: development and feasibility of a coding scheme. *Geriatric nursing, 36(2 Suppl),* S10–S15.	Focus is assessment not care
Gilmore-Bykovskyi, A. L., & Rogus-Pulia, N. (2018). Temporal associations between caregiving approach, behavioral symptoms and observable indicators of aspiration in nursing home residents with dementia. *The Journal of Nutrition Health and Aging, 22(3),* 400-406.	Not a primary study
Goddaer, J., & Abraham, I.L. (1994). Effects of relaxing music on agitation during meals among nursing home residents with severe cognitive impairment. Archives of Psychiatric Nursing, 8(3), 150-158.	Outside direct control of care staff
Hammar, L. M., Swall, A., & Meranius, M. S. (2016). Ethical aspects of caregivers' experience with persons with dementia at mealtimes. *Nursing Ethics, 23(6),* 624–635.	Focus is not mealtime care activities
Hanssen, I., & Kuven, B. M. (2016). Moments of joy and delight: The meaning of traditional food in dementia care. *Journal of Clinical Nursing, 25,* 866– 874.	Outside direct control of care staff
Harnett, T., & Jönson, H. (2017). Shaping nursing home mealtimes. *Ageing and Society, 37(4),* 823-844.	Population focus is not people with dementia
Hicks-Moore, S. L. (2005). Relaxing music at mealtimes in nursing homes: effects on agitated patients with dementia. Journal of Gerontological Nursing, 31(12), 26–32.	Outside direct control of care staff
Ho, S.-Y., Lai, H.-L., Jeng, S.-Y., Tang, C.-W., Sung, H.-C., & Chen, P.-W. (2011). The Effects of Researcher-Composed Music at Mealtime on Agitation in Nursing Home Residents With Dementia. Archives of Psychiatric Nursing, 25(6), e49-e55.	Outside direct control of care staff
Holdoway, A., & Smith, A. (2020). Meeting nutritional need and managing patients with dysphagia. Journal of *Community Nursing, 34*(2).	Population focus is not people with dementia
Holm, B., & Soderhamn, O. (2003). Factors associated with nutritional status in a group of people in an early stage of	Not all participants in care homes

dementia. Clinical Nutrition, 22(4), 385-389.	
Hsiao, H. C., Chao, H. C., & Wang J. J. (2013). Features of problematic eating behaviors among community-dwelling older adults with dementia: family caregivers' experience. Geriatric Nursing, 34(5), 361-365.	Not all participants in care homes
Hung, L., Chaudhury, H., & Rust, T. (2016). The Effect of Dining Room Physical Environmental Renovations on Person-Centered Care Practice and Residents' Dining Experiences in Long-Term Care Facilities. *Journal of Applied Gerontology, 35(12),* 1279–1301.	Population focus is not people with dementia
Hyden, L. C. (2014). Cutting Brussels sprouts: Collaboration involving people with dementia. *Journal of Aging Studies, 29,* 115–23.	Not all participants in care homes
Jansson, L., & Norberg, A. (1992). Ethical reasoning Among Registered Nurses Experienced in Dementia Care. Interviews Concerning the Feeding of Severely Demented Patients. *Scandinavian Journal of Caring Sciences, 6(4),* 219– 27.	Focus is not mealtime care activities
Jean, L. A. (1997). " Finger food menu" restores independence in dining. *Health care food & nutrition focus, 14(1),* 4-6.	Not peer-reviewed (missed on title/abstract screening)
Jensen, L. H., Rekve, K. H., Ulstein, I, & Skovdahl, K. (2016). Promoting independence at mealtimes for older persons with severe dementia. *International Practice Development Journal, 6(2),* 1-13.	Not all participants in care homes
Jimoh, O. F., Brown, T. J., Bunn, D., & Hooper, L. (2019). Beverage intake and drinking patterns—clues to support older people living in long-term care to drink well: DRIE and FISE studies. *Nutrients, 11*(2), 447.	Population focus is not people with dementia
Johansson, L., Wijk, H., & Christensson, L. (2017). Improving nutritional status of older persons with dementia using a national preventive care program. *Journal of Nutrition, health & aging, 21,* 292–298.	Not all participants in care homes
Johansson, L., Björklund, A., Sidenvall, B., & Christensson, L. (2017). Staff views on how to improve mealtimes for elderly people with dementia living at home. *Dementia, 16(7),* 835–852.	Not all participants in care homes
Jones, J. A., Brown E. J., & Ladislav, V. (2000). Target outcomes for long- term oral health care in dementia: a Delphi approach. *Journal of Public Health Dentistry, 60(4),* 330–333.	Outside direct control of care staff
Kayser-Jones J, Schell E. S., Porter C., Barbaccia J. C., & Shaw H. (1999). Factors contributing to dehydration in nursing homes: Inadequate staffing and lack of professional supervision. *Journal of the American Geriatrics Society, 47(10),* 1187–1194.	Outside direct control of care staff
Keller, H.H., Gibbs-Ward, A., Randall-Simpson, J., Bocock, M.-A., & Dimou, E. (2006). Meal rounds: An essential aspect of quality nutrition services in long-term care. *Journal of the American Medical Directors Association, 7(1),* 40-45.	Outside direct control of care staff
Keller, H. H., Edward, H. G., & Cook, C. (2007). Mealtime Experiences of Families With Dementia. *American Journal of Alzheimer's Disease & Other Dementias, 21(6),* 431–438.	Not all participants in care homes
Keller, H. H., Schindel Martin, L., Dupuis, S., Genoe, R., Gayle Edward, H., & Cassolato, C. (2010). Mealtimes and	Not all participants in care homes

being connected in the community-based dementia context. *Dementia, 9(2),* 191–213.	
Keller, H. H., Martin, L. S., Dupuis, S., Reimer, H., & Genoe, R. (2015). Strategies to support engagement and continuity of activity during mealtimes for families living with dementia; a qualitative study. *BMC Geriatrics, 15,* 119.	Not all participants in care homes
Keller, H. H., Carrier, N., Slaughter, S. E., Lengyel, C., Steele, C. M., Duizer, L., Morrison, J., Brown, K. S., Chaudhury, H., Yoon, M. N., Duncan, A. M., Boscart, V., Heckman, G., Villalon, L. (2017). Prevalence and Determinants of Poor Food Intake of Residents Living in Long-Term Care. *Journal of the American Medical Directors Association, 18(11),* 941-947.	Outside direct control of care staff
Kingston. T. (2017). Promoting fluid intake for patients with dementia or visual impairments. *British Journal of Nursing, 26(2),* 98–99.	Population focus is not people with dementia
Kontos, P. C., & Naglie, G. (2007). Bridging theory and practice: Imagination, the body, and person-centred dementia care. *Dementia, 6*(4), 549-569.	Focus is not mealtime care activities
Lee, K. M., & Song, J. A. (2012). Characteristics of Eating Behavior in Elders with Dementia residing in Long-Term Care Facilities. *Journal of Korean Academy of Nursing, 42(4),* 466-476.	Not in English (missed on title/abstract screening)
Lee, K. M., & Song, J. A. (2015). Factors influencing the degree of eating ability among people with dementia. *Journal of Clinical Nursing, 24, (11-12),* 1707-1717.	Focus is not mealtime care activities
Lea, E. J., Goldberg, L. R., Price, A. D., Tierney, L. T., & McInerney, F. (2017). Staff awareness of food and fluid care needs for older people with dementia in residential care: A qualitative study. *Journal of Clinical Nursing, 26(23-24),* 5169-5178.	Outside direct control of care staff
Lea, E. J., Goldberg, L. R., Price, A. D., Tierney, L. T., & McInerney FJ. (2019). Best intentions or best practice? A case study of the nutritional needs and outcomes of a person with dementia living in a residential aged care home. *International Journal of Nursing Practice, 25(1),* e12692.	Focus is not mealtime care activities
Littlewood, S., Saedi, S., & Williams, C. (1997). Mealtimes: a missed opportunity. *Journal of Dementia Care, July–August,* 18–21.	Not all participants in care homes
Liu, W., Galik, E., Nahm, E. S., Boltz, M., & Resnick, B. (2015). Optimizing eating performance for long-term care residents with dementia: Testing the impact of function-focused care for cognitively impaired. *Journal of the American Medical Directors Association, 16(12),* 1062-1068.	Focus is not mealtime care activities
Liu, W., Galik, E., Boltz, M., Nahm, E. S., Lerner, N., & Resnick, B. (2016). Factors associated with eating performance for long- term care residents with moderate- to- severe cognitive impairment. *Journal of advanced nursing, 72(2),* 348-360.	Focus is not mealtime care activities
Liu, W., Jao, Y. L., & Williams, K. (2017). The association of eating performance and environmental stimulation among older adults with dementia in nursing homes: A secondary analysis. *International journal of nursing*	Not a primary study

studies, 71, 70-79.	
Lopez, R. P., & Amella, E. J. (2011). Time travel: The lived experience of providing feeding assistance to a family member with dementia. *Research in gerontological nursing, 4(2),* 127-134.	Not all participants in care homes
Lou, M. F., Dai, Y. T., Huang, G. S., & Yu, P. J. (2007). Nutritional status and health outcomes for older people with dementia living in institutions. *Journal of advanced nursing, 60(5),* 470-477.	Focus is not mealtime care activities
Mamhidir, A. G., Karlsson, I., Norberg, A., & Mona, K. (2007). Weight increase in patients with dementia, and alteration in meal routines and meal environment after integrity promoting care. *Journal of clinical nursing, 16(5),* 987-996.	Outside direct control of care staff
Mann, K., Lengyel, C. O., Slaughter, S. E., Carrier, N., & Keller, H. (2019). Resident and Staff Mealtime Actions and Energy Intake of Long-Term Care Residents With Cognitive Impairment: Analysis of the Making the Most of Mealtimes Study. Journal of Gerontological Nursing, 45(8), 32-42.	Not a primary study
Manthorpe, J., Watson, R., & Stimpson, A. (2003). Cooking up a problem in the kitchen Changes in cooking, food preparation and eating habits may give rise to concern among relatives of people with dementia. *Journal of Dementia Care, 11(5),* 16-18.	Not all participants in care homes
Marsden, J. P., Meehan, R. A., & Calkins, M. P. (2001). Therapeutic kitchens for residents with dementia. *American Journal of Alzheimer's Disease & Other Dementias, 16(5),* 303-311.	Outside direct control of care staff
Martinsen, B., & Norlyk, A. (2012). Caregivers' lived experience of assisted feeding. *Journal of clinical nursing, 21(19pt20),* 2966-2974.	Population focus is not people with dementia
McDaniel, J. H., Hunt, A., Hackes, B., & Pope, J. F. (2001). Impact of dining room environment on nutritional intake of Alzheimer's residents: A case study. *American Journal of Alzheimer's Disease & Other Dementias, 16(5),* 297–302	Outside direct control of care staff
Milte, R., Shulver, W., Killington, M., Bradley, C., Miller, M., & Crotty, M. (2017). Struggling to maintain individuality–describing the experience of food in nursing homes for people with dementia. *Archives of Gerontology and Geriatrics, 72,* 52-58.	Not all participants in care homes
Milte, R., Ratcliffe, J., Chen, G., Miller, M., & Crotty, M. (2018). Taste, choice and timing: Investigating resident and carer preferences for meals in aged care homes. *Nursing & health sciences, 20(1),* 116-124.	Population focus is not people with dementia
Moore, D. (2003). The Feedback from Service Users Scale: views of people with dementia about their living environment. *Journal of Dementia Care, January–February,* 37–38.	Focus is not mealtime care activities
Niezgoda, H., Keller, H. H., Steele, C. M., & Chambers, L. W. (2014). What should a case-finding tool for dysphagia in long term care residents with dementia look like? *Journal of the American Medical Directors Association, 15(4),* 296-298.	Focus is assessment not care
Nolan, B. A., & Mathews, R. M. (2004). Facilitating resident information seeking regarding meals in a special care	Outside direct control of care staff

unit: an environmental design intervention. *Journal of Gerontological nursing, 30(10),* 12-16.	
Norberg, A., & Asplund, K. (1990). Caregivers' experience of caring for severely demented patients. *Western Journal of Nursing Research, 12(1),* 75-84.	Focus is not mealtime care activities
Norberg, A., & Hirschfield, M. (1987). Feeding of severely demented patients in institutions: interviews with caregivers in Israel. *Journal of advanced nursing, 12(5),* 551-557.	Focus is not mealtime care activities
Norberg, A., Norberg, B., & Bexell, G. (1980). Ethical problems in feeding patients with advanced dementia. *British Medical Journal,* 281, 847-848.	Not all participants in care homes
Norberg, A., Bäckström, Å., Athlin, E., & Norberg, B. (1988). Food refusal amongst nursing home patients as conceptualized by nurses' aids and enrolled nurses: an interview study. *Journal of Advanced Nursing, 13(4),* 478-483.	Focus is not mealtime care activities
Norberg, A., Hirschfeld, M., Davidson, B., Davis, A., Lauri, S., Lin, J. Y., ... & Ziv, L. (1994). Ethical reasoning concerning the feeding of severely demented patients: an international perspective. *Nursing Ethics, 1(1),* 3-13.	Focus is not mealtime care activities
Odencrants, S., Blomberg, K., & Wallin, A. M. (2020). "The meal is an activity involving at least two people"—Experiences of meals by older persons in need of elderly care. *Nursing Open, 7*(1), 265-273.	Not all participants in care homes
Osborn, C. L., & Marshall, M. J. (1993). Self-feeding performance in nursing home residents. *Journal of Gerontological Nursing, 19(3),* 7-9.	Focus is not mealtime care activities
Palese, A., Grassetti, L., Bressan, V., Decaro, A., Kasa, T., Longobardi, M., Hayter, M., & Watson, R. (2019). A path analysis on the direct and indirect effects of the unit environment on eating dependence among cognitively impaired nursing home residents. *BMC Health Services Research, 19*(1), 775.	Focus is not mealtime care activities
Palese, A., Gonella, S., Kasa, T., Caruzzo, D., Hayter, M., & Watson, R. (2019). Negative prompts aimed at maintaining eating independence. *Nursing ethics, 26*(7-8), 2158-2171.	Not a primary study
Papachristou, I., Giatras, N., & Ussher, M. (2013). Impact of dementia progression on food-related processes: a qualitative study of caregivers' perspectives. *American Journal of Alzheimer's Disease & Other Dementias, 28(6),* 568-574.	Not all participants in care homes
Pezzana, A., Cereda, E., Avagnina, P., Malfi, G., Paiola, E., Frighi, Z., ... & Amerio, M. L. (2015). Nutritional care needs in elderly residents of long-term care institutions: Potential implications for policies. *The journal of nutrition, health & aging, 19(9),* 947-954.	Focus is not mealtime care activities
Porter, C., Schell, E. S., Kayser-Jones, J., & Paul, S. M. (1999). Dynamics of nutrition care among nursing home residents who are eating poorly. *Journal of the American Dietetic Association, 99(11),* 1444-1446.	Outside direct control of care staff
Pouyet, V., Giboreau, A., Benattar, L., & Cuvelier, G. (2014). Attractiveness and consumption of finger foods in elderly Alzheimer's disease patients. *Food quality and preference, 34,* 62-69.	Outside direct control of care staff
Pouyet, V., Cuvelier, G., Benattar, L., & Giboreau, A. (2015). Influence of flavour enhancement on food liking and	Outside direct control of care staff

consumption in older adults with poor, moderate or high cognitive status. *Food Quality and Preference, 44,* 119-129.	
Ragneskog, H., Kihlgren, M., Karlsson, I., & Norberg, A. (1996). Dinner music for demented patients: analysis of video-recorded observations. *Clinical Nursing Research, 5(3),* 262-277.	Outside direct control of care staff
Ragneskog, H., Bråne, G., Karlsson, I., & Kihlgren, M. (1996). Influence of dinner music on food intake and symptoms common in dementia. *Scandinavian journal of caring sciences, 10(1),* 11-17.	Outside direct control of care staff
Richeson, N. E., & Neill, D. J. (2004). Therapeutic recreation music intervention to decrease mealtime agitation and increase food intake in older adults with dementia. *American Journal of Recreation Therapy, 3(1),* 37-41.	Outside direct control of care staff
Rivière, S., Gillette-Guyonnet, S., Nourhashemi, F., & Vellas, B. (1999). Nutrition and Alzheimer's disease. *Nutrition reviews, 57(12),* 363-367.	Not all participants in care homes
Roberts, S., & Durnbaugh, T. (2002). Enhancing nutrition and eating skills in long-term care. *Alzheimer's Care Today, 3(4),* 316-329.	Focus is not mealtime care activities
Roberts, E. (2011). Six for lunch: A dining option for residents with dementia in a special care unit. *Journal of Housing for the Elderly, 25(4),* 352-379.	Outside direct control of care staff
Salva, A., Coll-Planas, L., Bruce, S., De Groot, L., Andrieu, S., Abellan, G., & Vellas, B. (2009). Nutritional assessment of residents in long-term care facilities (LTCFs): recommendations of the task force on nutrition and ageing of the IAGG European region and the IANA. *The Journal of Nutrition, Health and Aging, 13(6),* 475-483.	Population focus is not people with dementia
Scott, D. (1999). Communication and swallowing training for care home staff. *Nursing And Residential Care, 1(6),* 318-321.	Population focus is not people with dementia
Shatenstein, B., Ska, B., & Ferland, G. (2001). Employee reactions to the introduction of a bulk food distribution system in a nursing home. *Canadian Journal of Dietetic Practice and Research, 62(1),* 18.	Outside direct control of care staff
Silva, P., Kergoat, M. J., & Shatenstein, B. (2013). Challenges in managing the diet of older adults with early-stage Alzheimer dementia: a caregiver perspective. *The journal of nutrition, health & aging, 17(2),* 142-147.	Not all participants in care homes
Simmons, S. F., Lam, H. Y., Rao, G., & Schnelle, J. F. (2003). Family members' preferences for nutrition interventions to improve nursing home residents' oral food and fluid intake. *Journal of the American Geriatrics Society, 51(1),* 69-74.	Outside direct control of care staff
Simmons, S. F., Coelho, C. S., Sandler, A., & Schnelle, J. F. (2018). A quality improvement system to manage feeding assistance care in assisted-living. *Journal of the American Medical Directors Association, 19(3),* 262-269.	Outside direct control of care staff
Slaughter, S. E., Morrison-Koechl, J. M., Chaudhury, H., Lengyel, C. O., Carrier, N., & Keller, H. H. (2020). The association of eating challenges with energy intake is moderated by the mealtime environment in residential	Focus is not mealtime care activities

care homes. International Psychogeriatrics, 1-11.	
Steele, C. M., Rivera, T., Bernick, L., & Mortensen, L. (2007). Insights regarding mealtime assistance for individuals in long-term care: lessons from a time of crisis. *Topics in Geriatric Rehabilitation, 23(4),* 319-329.	Population focus is not people with dementia
Stockdell, R., & Amella, E. J. (2008). How to Try This: The Edinburgh Feeding Evaluation in Dementia Scale. *American Journal of Nursing, 108(8),* 46.	Not all participants in care homes
Suominen, M. H., Kivisto, S. M., & Pitkala, K. H. (2007). The effects of nutrition education on professionals' practice and on the nutrition of aged residents in dementia wards. *European journal of clinical nutrition, 61(10),* 1226-1232.	Focus is not mealtime care activities
Tarzian, A. (2019). Foregoing Spoon Feeding in End-Stage Dementia. The American Journal of Bioethics: AJOB, 19(1), 88-89.	Not a primary study
Thomas, D. W., & Smith, M. (2009). The effect of music on caloric consumption among nursing home residents with Dementia of the Alzheimer's type. *Activities, Adaptation & Aging, 33(1),* 1-16.	Outside direct control of care staff
Törmä, J., Winblad, U., Saletti, A., & Cederholm, T. (2018). The effects of nutritional guideline implementation on nursing home staff performance: A controlled trial. *Scandinavian journal of caring sciences, 32(2),* 622-633.	Population focus is not people with dementia
van Hoof, J., Verbeek, H., Janssen, B. M., Eijkelenboom, A., Molony, S. L., Felix, E., ... & Wouters, E. J. M. (2016). A three perspective study of the sense of home of nursing home residents: the views of residents, care professionals and relatives. *BMC Geriatrics, 16(1),* 169.	Population focus is not people with dementia
Van Ort, S., & Phillips, L. R. (1995). Nursing interventions to promote functional feeding. *Journal of gerontological nursing, 21(10),* 6-9.	Population focus is not people with dementia
Watson, R., & Deary, I. (1996). Is there a relationship between feeding difficulty and nursing intervention in elderly people with dementia? *Journal of Research in Nursing, 1(1),* 44-54.	Focus is not mealtime care activities
Watson, R., & Deary, I. J. (1997). A longitudinal study of feeding difficulty and nursing intervention in elderly patients with dementia. *Journal of Advanced Nursing, 26(1),* 25-32.	Focus is not mealtime care activities
Watson, R. (1994). Measuring feeding difficulty in patients with dementia: replication and validation of the EdFED Scale# 1. Journal of Advanced Nursing, 19(5), 850-855.	Focus is assessment not care
Wong, F., Keller, H. H., Schindel Martin, L., & Sutherland, O. (2015). A recipe for mealtime resilience for families living with dementia. *Scandinavian journal of caring sciences, 29(3),* 486-494.	Not all participants in care homes
Volkert, D., Chourdakis, M., Faxen-Irving, G., Frühwald, T., Landi, F., Suominen, M. H., ... & Schneider, S. M. (2015). ESPEN guidelines on nutrition in dementia. *Clinical nutrition, 34(6),* 1052-1073.	Not a primary study
Young, K. W., Binns, M. A., & Greenwood, C. E. (2001). Meal delivery practices do not meet needs of Alzheimer patients with increased cognitive and behavioral difficulties in a long-term care facility. *The Journals of Gerontology Series A: Biological Sciences and Medical Sciences, 56(10),* M656-M661.	Outside direct control of care staff

Supplementary Table A. Study quality – quantitative studies

Critical appraisal question	Study	
Analytical cross sectional studies	Suski & Nielsen (1989)	Amella (1999)
Were the criteria for inclusion in the sample clearly defined?	Y	Y
Were the study subjects and the setting described in detail?	Y	Y
Was the exposure measured in a valid and reliable way?	N/A	Y
Were objective, standard criteria used for measurement of the condition?	U	Y
Were confounding factors identified?	Y	N
Were strategies to deal with confounding factors stated?	U	Y
Were the outcomes measured in a valid and reliable way?	N	Y
Was appropriate statistical analysis used?	Y	Y
Quasi-experimental studies	Engström & Hammar (2012)	
Is it clear in the study what is the 'cause' and what is the 'effect' (i.e. there is no confusion about which variable comes first)?	Y	
Were the participants included in any comparisons similar?	Y	
Were the participants included in any comparisons receiving similar treatment/care, other than the exposure or intervention of interest?	Y	
Was there a control group?	N	
Were there multiple measurements of the outcome both pre and post the intervention/exposure?	Y	
Was follow up complete and if not, were differences between groups in terms of their follow up adequately described and analyzed?	N	
Were the outcomes of participants included in any comparisons measured in the same way?	Y	
Were outcomes measured in a reliable way?	Y	
Was appropriate statistical analysis used?	N	
Randomized Controlled Trials	Coyne and Hoskins (1997)	Batchelor-Murphy et al. (2017)

Was true randomization used for assignment of participants to treatment groups?	Y	U
Was allocation to treatment groups concealed?	Y	N/A
Were treatment groups similar at the baseline?	N	N/A
Were participants blind to treatment assignment?	U	N/A
Were those delivering treatment blind to treatment assignment?	N/A	N/A
Were outcomes assessors blind to treatment assignment?	Y	N/A
Were treatment groups treated identically other than the intervention of interest?	Y	Y
Was follow up complete and if not, were differences between groups in terms of their follow up adequately described and analyzed?	Y	Y
Were participants analyzed in the groups to which they were randomized?	Y	Y
Were outcomes measured in the same way for treatment groups?	Y	Y
Were outcomes measured in a reliable way?	Y	Y
Was appropriate statistical analysis used?	Y	Y
Was the trial design appropriate, and any deviations from the standard RCT design (individual randomization, parallel groups) accounted for in the conduct and analysis of the trial?	U	Y

Questions are from the Joanna Briggs Institute Critical Appraisal Tools. Y=Yes, N=No, U=Unclear, N/A=Not applicable.

Supplementary Table B. Study quality – qualitative studies

**Critical appraisal question**	**Study**												
	Van Ort & Phillips (1992)	Kayser-Jones & Schell (1997a)	Kayser-Jones & Schell (1997b)	Pierson (1999)	De Bellis et al. (2003)	Pasman et al. (2003)	Gibbs-Ward & Keller (2005)	Chang & Roberts (2008)	Hung & Chaudhury (2011)	Nell et al. (2016)	Murphy et al. (2017)	Palese et al. (2018)	Driessen & Ibáñez Martín (2020)
Is there congruity between the stated philosophical perspective and the research methodology?	Y	U	U	Y	Y	N	Y	U	Y	U	U	N	U
Is there congruity between the research methodology and the research question or objectives?	Y	Y	Y	Y	Y	Y	Y	U	Y	Y	Y	U	Y

Is there congruity between the research methodology and the methods used to collect data?	Y	Y	Y	Y	Y	Y	Y	U	Y	Y	Y	N	Y
Is there congruity between the research methodology and the representation and analysis of data?	Y	Y	U	Y	Y	Y	Y	U	Y	Y	Y	N	U
Is there congruity between the research methodology and the interpretation of results?	Y	Y	Y	Y	Y	Y	Y	U	Y	Y	Y	N	U
Is there a statement locating the researcher culturally or theoretically?	Y	Y	Y	Y	Y	Y	Y	U	Y	Y	Y	N	Y
Is the influence of the researcher on the research, and vice-versa, addressed?	U	N	Y	U	N	U	Y	N	N	N	U	Y	N
Are participants, and their voices, adequately represented?	U	N	N	U	N	Y	Y	N	Y	N	U	Y	U
Is the research ethical according to current criteria or, for recent studies, is there evidence of ethical approval by an appropriate body?	N	Y	U	Y	N	Y	Y	N	N	Y	Y	Y	Y
Do the conclusions drawn in the research report flow from the analysis, or interpretation, of the data?	N	N	N	Y	N	Y	Y	Y	Y	Y	Y	Y	U

Questions are from the Joanna Briggs Institute Critical Appraisal Tools. Y=Yes, N=No, U=Unclear, N/A=Not applicable.
